# Long‐Term Biobanked Dental Pulp Stem Cells Retain Angiogenic Potential for Vascularised Tissue Engineering—Laboratory Investigation

**DOI:** 10.1111/iej.70036

**Published:** 2025-09-23

**Authors:** Shuntaro Yamada, Katerina Holomkova, Åshild Johansen, Masoumeh Jahani Kadousaraei, Niyaz Al‐Sharabi, Francesco Torelli, Pierfrancesco Pagella, Ana Angelova Volponi, Hiroshi Egusa, Inge Fristad, Kamal Mustafa

**Affiliations:** ^1^ Center of Translational Oral Research ‐ Tissue Engineering, Department of Clinical Dentistry, University of Bergen Bergen Norway; ^2^ Centre for Craniofacial & Regenerative Biology, Faculty of Dentistry, Oral & Craniofacial Sciences, King's College London London UK; ^3^ Department of Histology and Embryology Faculty of Medicine, Masaryk University Brno Czechia; ^4^ Institute of Animal Physiology and Genetics Czech Academy of Sciences Brno Czechia; ^5^ Laboratory of Molecular Materials, Division of Biophysics and Bioengineering, Department of Physics Chemistry and Biology (IFM), Linköping University Linköping Sweden; ^6^ Center for Advanced Stem Cell and Regenerative Research, Tohoku University Graduate School of Dentistry Miyagi Japan; ^7^ Division of Molecular & Regenerative Prosthodontics, Tohoku University Graduate School of Dentistry Miyagi Japan

**Keywords:** angiogenesis, dental pulp, dental research, mesenchymal stem cells, regenerative medicine, tissue engineering

## Abstract

**Aim:**

This study aimed to evaluate whether human dental pulp stem cells (DPSCs), after long‐term biobanking (7–8 years), retain their pro‐angiogenic properties and can be used to engineer vascularised tissues, addressing their potential for clinical translation in regenerative dentistry.

**Methodology:**

Cryopreserved DPSCs from adolescent donors were recovered from biobanking and characterised for chromosomal integrity, MSC immunophenotype and multipotency. After conditioning in pro‐angiogenic conditions in vitro, gene and protein expression were analysed by RT‐qPCR array, flow cytometry and high‐throughput immunophenotyping. Functional angiogenic capacity was assessed via in vitro tube formation, *ex ovo* CAM implantation assay, organ‐on‐chip perfusion model and long‐term culture (45 days) in clinical‐grade GelMA hydrogels, with and without HUVECs.

**Results:**

Biobanked DPSCs retained MSC identity and multi‐lineage differentiation potential. Pro‐angiogenic/endothelial conditioning enhanced the expression of angiogenic/endothelial genes (PECAM1, VEGFR2, NRP1, ACE), yet most cells maintained a pericyte‐like phenotype. Both naive and endothelial‐conditioned DPSCs (i.e., naiveDPSCs and endoDPSCs, respectively) significantly enhanced vascular ingrowth in the CAM model. In the organ‐on‐chip system, naiveDPSCs formed perfusable vasculature with HUVECs and differentiated into perivascular cell types. Most notably, endoDPSCs alone successfully generated vascularised tissue with both CD31(+) and αSMA(+) cells present in GelMA hydrogels after prolonged stimulation.

**Conclusion:**

Long‐term biobanked DPSCs preserve their angiogenic potential and, following extended endothelial induction, can independently generate vascularised tissue in 3D in vitro culture models. This is the first report demonstrating the comprehensive pro‐angiogenic characterisation and the feasibility of using biobanked DPSCs for vascularised tissue engineering, highlighting their strong clinical applicability for future regenerative therapies.

## 1 Introduction

Dental pulp stem cells (DPSCs) are mesenchymal stem/stromal cells (MSCs) isolated from the dental pulp. In regenerative medicine, DPSCs have gained significant attention due to their accessibility and multipotency (Egusa et al. 2012). Among their diverse capabilities, their pro‐angiogenic properties stand out as particularly valuable for regenerative therapies requiring vascularisation, such as those targeting ischaemic tissues or vascularised tissue engineering.

As prominence of cell‐based regenerative therapies becomes recognised, public interest in dental stem cell biobanking has grown. In response, academic institutions and commercial entities have established dental stem cell biobanking services worldwide, offering clinical‐grade cell manufacturing and storage (Yamada et al. 2025). Patients opt to preserve these cells at the time of tooth exfoliation or extraction for various reasons, anticipating future advancements in cell therapy for regenerative medicine and dentistry. It is generally accepted that cryopreservation preserves cellular profiles after recovery, as evidenced by their retention of multipotency, viability and immunophenotype (Bahsoun et al. 2019; Shorokhova et al. 2024; Wang and Li 2024). However, recent studies have suggested potential genetic, epigenetic and transcriptional shifts after biobanking depending on cell types and duration (Shorokhova et al. 2024; Thaler et al. 2012), resulting potentially in compromised regenerative capacities. Concerning DPSCs, there is particular unclarity when it comes to the preservation of their pro‐angiogenic capabilities upon prolonged biobanking. Extended cryopreservation may alter their regenerative potential, particularly in vascular‐related applications, necessitating further investigation.

Angiogenesis is critical for maintaining oxygen and nutrient supply during tissue regeneration (Liu et al. 2021). With dental stem cells, this is believed to occur through their direct differentiation into vascular endothelial cells, via secretion of pro‐angiogenic factors and/or their perivascular localisation and support akin to pericytes. For example, in the presence of vascular endothelial growth factor (VEGF) and/or endothelial growth medium, DPSCs express endothelial markers, such as CD31 and von Willebrand factor (vWF), and form vasculature‐like structures both in vitro and in vivo (Bento et al. 2013; Itoh et al. 2018; Janebodin et al. 2013; Katata et al. 2021; Ma et al. 2024; Sasaki et al. 2020). However, despite their reported potential, their detailed endothelial and pro‐angiogenic profiles remain underdocumented, with inconstant, if not conflicting, findings attributed to variations in cell sources, experimental designs and characterisation methods (Ganapathy et al. 2024; Sasaki et al. 2020; Tenyi et al. 2023). In particular, while most reported studies utilised either freshly isolated cells, cells purchased from companies for research, or rodent‐derived cells, no studies have tested the pro‐angiogenic properties of human dental stem cells following long‐term biobanking.

In the present study, we aim to evaluate the retained functionality of human biobanked DPSCs for applications in vascularised tissue engineering. Human DPSCs from adolescent donors following 7–8 years of biobanking (defined as long‐term biobanking in the present study) were firstly characterised by high‐throughput phenotyping donor‐individually and functional analysis under pro‐angiogenic/endothelial conditions as well as in *ex ovo* implantation in a chicken embryo model. Furthermore, their utility for vascularised tissue engineering was tested using a microfluidic organ‐on‐chip model and prolonged 3D culture in clinical‐grade hydrogels. These approaches provide a comprehensive characteristic of biobanked‐DPSC behaviours for angiogenesis and new insights into the translational potential of biobanked DPSCs.

## 2 Materials and Methods

**FIGURE 1:**
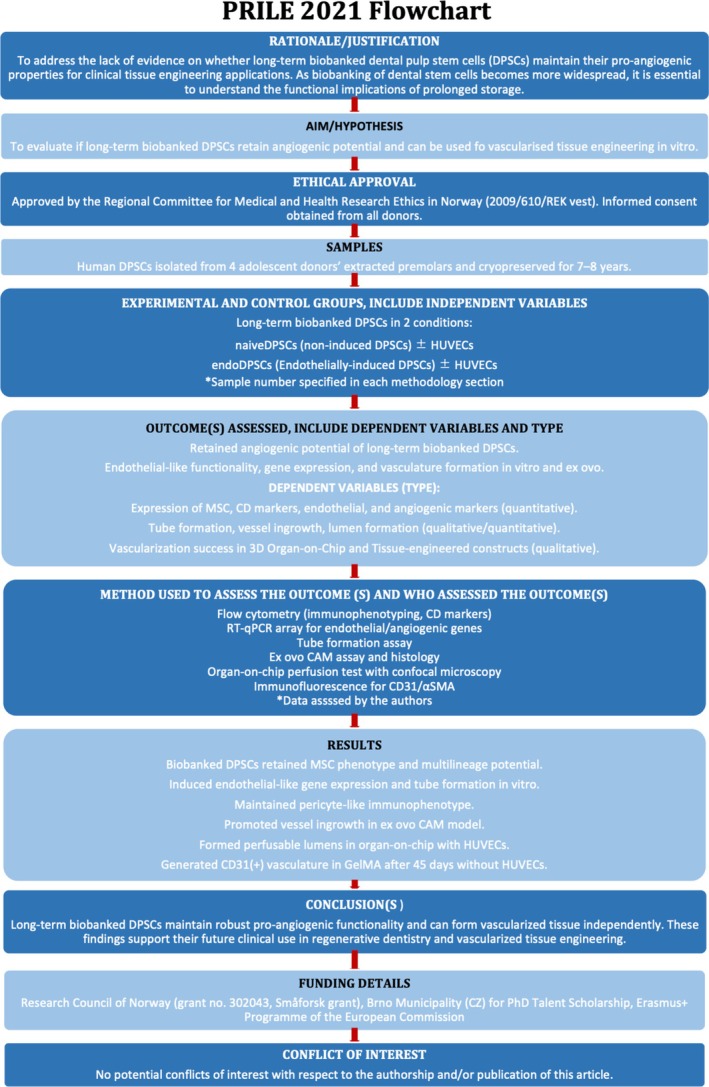
PRILE 2021 flowchart outlining the methodology and principal findings involved in this study.

This laboratory study has been reported in accordance with the Preferred Reporting Items for Laboratory Studies in Endodontology (PRILE) 2021 guidelines (Nagendrababu et al. 2021), with the research methodology and principal findings structured accordingly (Figure 1).

### 2.1 DPSC Isolation and Biobanking

The use of human samples was approved by the Regional Committee for Medical and Health Research Ethics in Norway (2009/610/REK vest). Dental pulps were collected from the premolars of 4 donors, aged between 18 and 24 years, after informed consent. All collected teeth had closed apices and were free of caries, with no signs of inflammation or pathology in either the pulp or periodontal tissues.

The tissue was enzymatically digested using collagenase type 1 (4 mg/mL) and dispase (2 mg/mL) for 1 h at 37°C before being plated into cell culture flasks. DPSCs were cultured in growth medium containing Dulbecco's Modified Eagle's Medium (DMEM; 10566016, Gibco, USA), supplemented with 10% foetal bovine serum (FBS; 10270‐106, Gibco, USA) and 1% penicillin/streptomycin (SV30010, HyClone, USA) at 37°C and 5% CO_2_ in a humidified incubator. At passage 1, the cells were cryopreserved donor‐independently in a standardised protocol. DPSCs were detached using Trypsin–EDTA (ECM0920D, Euroclone) and centrifuged at 300 rcf for 5 min. Cells were then resuspended in 10% dimethyl sulfoxide (DMSO), 20% FBS, 70% DMEM and subjected to a controlled cooling rate of 1°C per hour using a Mr. Frosty freezing container at −80°C for 72 h. Subsequently, the cells were stored in liquid nitrogen for 7–8 years stably until use. DPSCs at passage 3–5 were used for the study. DPSCs from 4 donors were pooled at passage 3 to average the DPSC phenotype and functionality, while high‐throughput profiling and characterisation were donor‐independently assessed to capture potential donor variation.

### 2.2 MSC Characterisation and Multilineage Differentiation

Post‐thaw, DPSCs from each donor were individually assessed for chromosomal integrity and normality using G‐banding karyotyping (data not shown). MSC characterisation was performed through immunophenotyping by flow cytometry and evaluation of multi‐lineage differentiation potential.

Immunophenotyping was conducted by flow cytometry against positive and negative MSC markers defined by the International Society for Cell & Gene Therapy (ISCT) (Dominici et al. 2006), in addition to Axin2, Sox2, c‐kit (CD117) and Stem Cell Factor (SCF). For the assessment of their multilineage differentiation, DPSCs were induced towards osteogenic, adipogenic, chondrogenic and neurogenic lineages (Appendix).

### 2.3 Induction of Endothelial Differentiation of DPSCs

Endothelial differentiation was optimised using 5 different medium compositions based on previously employed protocols (Bergamo et al. 2021; Janebodin et al. 2013; Li et al. 2023; Sasaki et al. 2020) while the growth medium served as a control. These media include (1) DMEM with 10% FBS (referred to as naiveDPSCs), (2) EGM‐2 medium (EGM2; C3162, Lonza, Switzerland), (3) EGM‐MV2 medium (EGMMV2; C22022, PromoCell, Germany), (4) DMEM with 2% FBS and 50 ng/mL recombinant human VEGF165 (293‐VE‐50, Bio‐Techne, USA), (5) EGM2 with 50 ng/mL VEGF165 (referred to as endoDPSCs) and (6) EGMMV2 with 50 ng/mL VEGF165. The initial seeding density of 10 000 cells/cm^2^ was applied.

### 2.4 Real‐Time Monitoring and Analysis of Growth Kinetics and Migration During Endothelial Induction

For real‐time monitoring of DPSCs after 10 days of endothelial induction, the 500 cells were re‐seeded in a 96‐well plate and incubated overnight at 37°C in a 5% CO_2_ humidified atmosphere (*n* = 12/group). On the following day, the plates were placed in a live‐imaging microscope (IncuCyte S3, Sartorius, Germany). Phase contrast images were acquired by a 10× objective every 2 h for 3 days until the cells became confluent. Cell confluency was analysed using the IncuCyte Base Analysis Software.

Likewise, their migration was evaluated by scratch wound healing assay in real time (*n* = 12/group). Standardised scratches in the width of 700–800 μm were generated using IncuCyte 96‐Well Wound Maker in accordance with the manufacturer's protocol. During the wound healing assay, cell proliferation was inhibited by 2.0 μg/mL aphidicolin (HY‐N6733, MedChemExpress, USA) in dimethyl sulfoxide (DMSO) to eliminate the effect of their different proliferation capacities. Images were acquired hourly for 24 h until complete wound closure and analysed in the IncuCyte software.

Cell metabolism of DPSCs after 10 days of endothelial induction was measured through Resazurin reduction using PrestoBlue assay (A13261, Invitrogen, USA) in accordance with the manufacturer's protocol (*n* = 12/group). In brief, the cells were seeded in 96‐well plates at a density of 5000 cells/cm^2^ and incubated overnight in a respective medium. The cells were then incubated with PrestoBlue‐medium solution for 30 min at 37°C in a 5% CO_2_ humidified atmosphere. Fluorescence was measured at ex/em = 560/590 nm using a microplate reader (Varioskan LUX multimode, VLBL00D0, Thermo Scientific, USA).

### 2.5 Flow Cytometry

The expression of MSC markers, 5 pericyte and 5 endothelial markers, as well as NOTCH1‐4 expression, was evaluated by flow cytometry. Experimental procedures, as well as antibodies and isotype controls used for the analysis, are provided in the Appendix S1 and Table S1, respectively.

### 2.6 Immunofluorescence and Confocal Microscopy

Samples were fixed in 4% PFA for 15 min followed by permeabilisation in 0.1% Triton X‐100 in PBS for 15 min and blocking in 10% normal goat serum for 60 min. The samples were then incubated with the Alexa Fluor 488 anti‐αSMA antibody (1:250; 53‐9760‐82, Invitrogen, USA) and CoraLite594 anti‐human PECAM1 antibody (1:200; CL594‐66065, ProteinTech, USA) overnight at 4°C. The nuclei were counterstained with Hoechst 33342 (B2261, Merck, Germany).

The z‐stack images were captured by a confocal microscope (Dragonfly 505, Andor Technologies, UK) equipped with 20× and 40× objectives and displayed as maximum projection images generated by Fiji/ImageJ or 3D rendered images by Imaris software (Oxford Instruments, UK).

### 2.7 RT‐qPCR Array for Endothelial and Angiogenic Gene Expression

To evaluate endothelial characteristics of induced DPSCs, a custom array of RT‐qPCR was designed based on the TaqMan system (4413257, Applied Biosystems, USA). It included 43 markers involved in endothelial differentiation and angiogenesis and five housekeeping genes. The list of genes and assay IDs is provided in Table S2. RNA extraction, cDNA preparation, and RT‐qPCR reactions were conducted as previously described (Yamada et al. 2024). Samples from 4 donors were individually evaluated (*n* = 4/group).

### 2.8 Tube Formation Assay

DPSCs induced for endothelial differentiation for 10 days were seeded on growth factor‐reduced (GFR) basement membrane matrix (Matrigel GFR, LDEV‐free; 356 231, Corning, USA) in a μ‐Slide 15 well 3D (10 000 cells per well; 81 506, ibidi, Germany) (*n* = 4/group). After incubation for 3 h in the corresponding medium, images were acquired with a 4× objective and analysed using the Angiogenesis Analyser for ImageJ.

### 2.9 CD Surface Marker Profiling and Bioinformatics

High‐throughput CD marker profiling was conducted using the SocioCD antibody‐based microarray (Sciomics, Germany) testing 184 CD markers. The data was acquired from samples of four donors in both naiveDPSC and endoDPSC groups (*n* = 4/group). Sample preparation and data acquisition were described in Appendix S1 and Tables S3 and S4. Bioinformatics was performed with STRING v12 and g:Profiler for Protein–Protein Interaction and Gene Ontology (GO) term enrichment, respectively.

### 2.10 Chorioallantoic Membrane Assay and Vessel Quantification

Chorioallantoic membrane (CAM) assay was conducted using *Gallus gallus domesticus* as previously described (Yamada et al. 2024). After 5 days of cell implantation, angiogenesis and vasculogenesis were evaluated by vessel quantifications and histological observations (final *n* = 9–11/group). The detailed experimental procedure is provided in the Appendix S1.

### 2.11 Vasculogenesis on Microfluidic Organ‐oChip

To evaluate the capacity of forming functional vascularised tissues, a microfluidic organ‐on‐chip (idenTx 3, AIM Biotech, Singapore) was employed (*n* = 4–6/group). The sample groups were: endoDPSCs, human umbilical vein endothelial cells (HUVECs), and naiveDPSCs co‐cultured with human umbilical vein endothelial cells (HUVECs) at a 1:5 ratio as preliminarily optimised. Fibrin hydrogel was prepared by mixing the equal volume of cell‐containing 5 mg/mL fibrinogen solution (F8630, Sigma‐Aldrich, USA) and 4 U/mL thrombin solution (T7513, Sigma‐Aldrich, USA). A total of 600 000 cells were encapsulated in 10 μL of fibrin hydrogel injected into a hydrogel channel.

Gravity‐driven interstitial flow was generated as previously described by applying 90 μL of EGM2 on one side of the medium channel and 50 μL on the other, with the medium refreshed daily. After 3 days of incubation, HUVECs were seeded in the medium channels to enhance perfusability. After 6 days, vasculogenesis was evaluated microscopically. The cells were incubated with Calcein AM (1:200; L3224, Invitrogen, USA) for 20 min, after which 10 μL of MiliQ water containing red fluorescent‐conjugated microbeads with a diameter of 4 μm (1:2000; F8858, ThermoFisher, USA) was loaded into the upstream region of the medium channels. The microfluidic chips were then placed on the Dragonfly 505 microscope, and z‐stack images were acquired every 7 s for 3 min. Additionally, a TRITC‐Dextran 70 kDa solution (TdB Labs, Sweden) was perfused to assess watertightness.

### 2.12 Vascularised Tissue Engineering Using GelMA Hydrogel

Vascularised tissue engineering was performed using naiveDPSCs and endoDPSCs, with and without HUVECs (*n* = 6/group). The cells were encapsulated at a density of 10 million cells/mL in a 5% (w/v) gelatin methacryloyl (GelMA; X‐Pure GelMA 160P60 RG, Rousselot Biomedical, Belgium) supplemented with 0.5% (w/v) LAP (L0290, Tokyo Chemical Industries, Japan). 80 μL of cell‐containing GelMA precursor was then crosslinked by high‐energy visible light at a light intensity of 1200 mW/cm^2^ as described previously (Yamada et al. 2024). The cell‐laden hydrogels were cultured in either DMEM with 10% FBS or EGM2 with 50 ng/mL VEGF for 35 days.

### 2.13 Statistics

All qualitative experiments were repeated a minimum of 3 times, and biological replicates (either pooled DPSCs or donor‐individual DPSCs) were individually plotted in the graphs. Unless stated otherwise, all data are presented as mean ± SEM. Statistical analyses, if not specified otherwise, were conducted using Prism 9 (Dotmatics, USA). For multiple comparisons, one‐way analysis of variance (ANOVA) followed by Tukey's multiple comparisons was applied. For pairwise comparisons, an unpaired Student's *t*‐test was used. A *p*‐value of < 0.05 was considered statistically significant.

## 3 Results

### 3.1 MSC Immunophenotype and Multipotency Retained in Long‐Term Biobanked DPSCs

**FIGURE 2:**
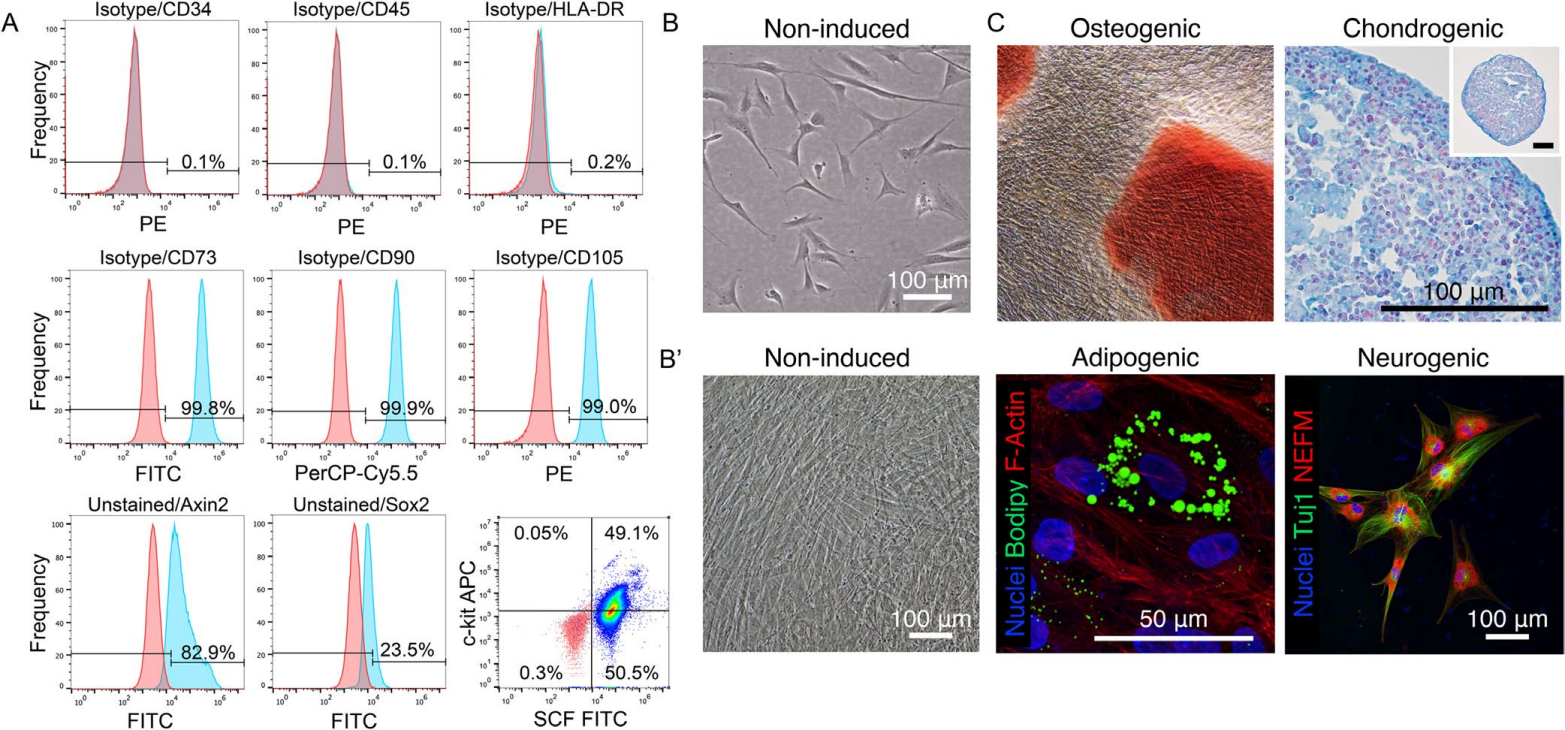
MSC marker characterisation and multilineage differentiation of DPSCs biobanked for long‐term. (A) DPSCs expressed MSC positive markers CD73, CD90, and CD105 exclusively (> 99%) but not negative markers, CD34, CD45, and HLA‐DR (< 0.5%). In addition, they expressed, but not exclusively, Axin2, Sox2, c‐kit, and Stem cell factor (SCF), suggesting their heterogeneity of cell population. (B, B′) Microscopic image of cell morphology at low and high confluency. (C) Biobanked DPSCs retained their multipotency, capable of differentiating into osteogenic (Alizarin Red S staining), chondrogenic (Alcian Blue staining), adipogenic (confocal microscopic image of the cells stained for fat droplets) and neurogenic (confocal microscopic image of the cells stained for Tuj1 and Neurofilament‐M [NEFM]) lineages.

Following to long‐term biobanking, DPSCs from all the donors satisfied MSC criteria where over 95% of the cells expressed positive markers, CD73, CD90 and CD105 while lacking the expression of negative markers, CD34, CD45 and HLA‐DR. In addition, the flow cytometry identified the presence of a subpopulation, namely Axin2‐, Sox2‐, c‐kit (CD117)‐ and SCF‐expressing DPSCs (Figure 2A).

The biobanked DPSCs retained spindle‐like morphology, which was able to differentiate into osteo/odontoblasts, chondrocytes, adipocytes and neuronal‐like cells upon the inductive stimuli (Figure 2B).

### 3.2 Endothelial‐Like Phenotype and mRNA Expression Profiles of DPSCs

**FIGURE 3:**
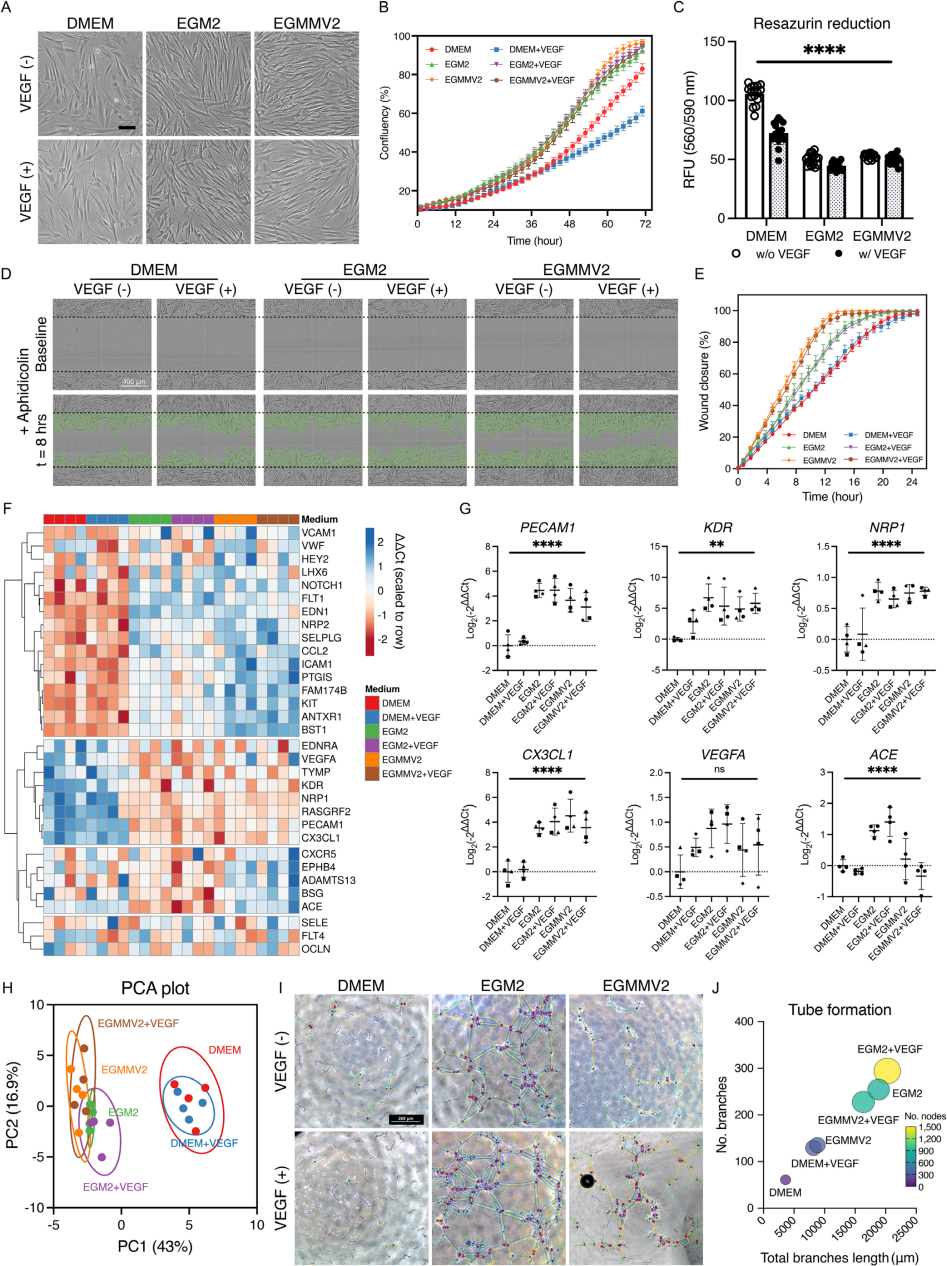
In vitro DPSC response to pro‐angiogenic conditions. (A) Morphology of DPSCs cultured under six different pro‐angiogenic conditions. (B) Real‐time growth monitoring of DPSCs. (C) Metabolic activity measurement of DPSCs (demonstrated as mean ± SD). (D, E) Wound scratch healing assay and real‐time monitoring of wound closure. (F) Heatmap image of tailored qRT‐PCR array targeting 43 markers related do endothelial cells and angiogenesis normalised against 5 house‐keeping genes. 32 markers were statistically defined as differentially expressed genes (DEG). DPSCs from 4 donors were individually assessed. (G) PCA plot showing the distribution of DPSCs cultured under the different conditions. Each dot represents DPSCs from individual donor. (H) Gene expression of key 6 endothelial and angiogenesis‐related markers. (I, J) Tube formation assays using DPSCs under the pro‐angiogenic conditions. Scale bar (black) = 100 μm. **p < 0.01, ****p < 0.0001.

To identify a suitable pro‐angiogenic condition in vitro, common endothelial stimulative medium reported for inducing endothelial‐like DPSC phenotype was compared. Under such conditions, DPSCs exhibited more elongated morphologies (Figure 3A). When EGM2 or EGMMV2 media were used, regardless of the presence of VEGF, cell proliferation was significantly promoted (Figure 3B). On the contrary, the DMEM with 2% FBS and VEGF group showed a lower growth rate, likely due to the reduced FBS concentration compared to the growth medium control (i.e., DMEM with 10% FBS).

After 10 days of conditioning, cells cultured in the pro‐angiogenic condition showed reduced metabolic activity compared to the control (Figure 3C). However, the wound scratching assay indicated that induced DPSCs, particularly by EGMMV2 with/without VEGF, closed the wound faster, suggesting the enhanced migration capacity of the cells (Figure 3D,E).

To assess the alteration of gene expression pattern, 43 markers related to angiogenesis and vascular endothelial differentiation were tested by the tailored RT‐qPCR array, in which 32 among 43 tested markers (Table S2) were differentially expressed at an mRNA level (Figure 3F). Compared to the control, both media induced a distinct expression profile of endothelial and angiogenic markers, with a marginal additional effect from VEGF (Figure 3G). Notably, both groups displayed similar profiles, except for CXCR5, EPHB4, ADAMTS13, BSG and ACE, which were upregulated only with EGM2 with/without VEGF. Regardless of VEGF supplementation, key endothelial and angiogenic markers, including PECAM1 (*p* < 0.0001), KDR (VEGFR2) (*p* < 0.0001), NRP1 (*p* < 0.001), ACE (*p* < 0.0001) and CX3CL1 (*p* < 0.0001) were significantly upregulated in both EGM2 and EGMMV2 groups despite donor variability (Figure 3H). Tube formation assays confirmed endothelial‐like phenotypes in DPSCs cultured in the endothelial media, with the densest honeycombed structure observed in the EGM2 + VEGF group (Figure 3I,J). Taken together, the EGM2 + VEGF group was identified as the most suitable in vitro pro‐angiogenic condition and therefore utilised for the subsequent study. DPSCs cultured under the condition are named endoDPSCs in comparison to naiveDPSCs (i.e., DPSCs cultured in the growth medium).

### 3.3 Predominance of Pericyte Phenotype With Pro‐Angiogenic Characteristics in endoDPSCs

**FIGURE 4:**
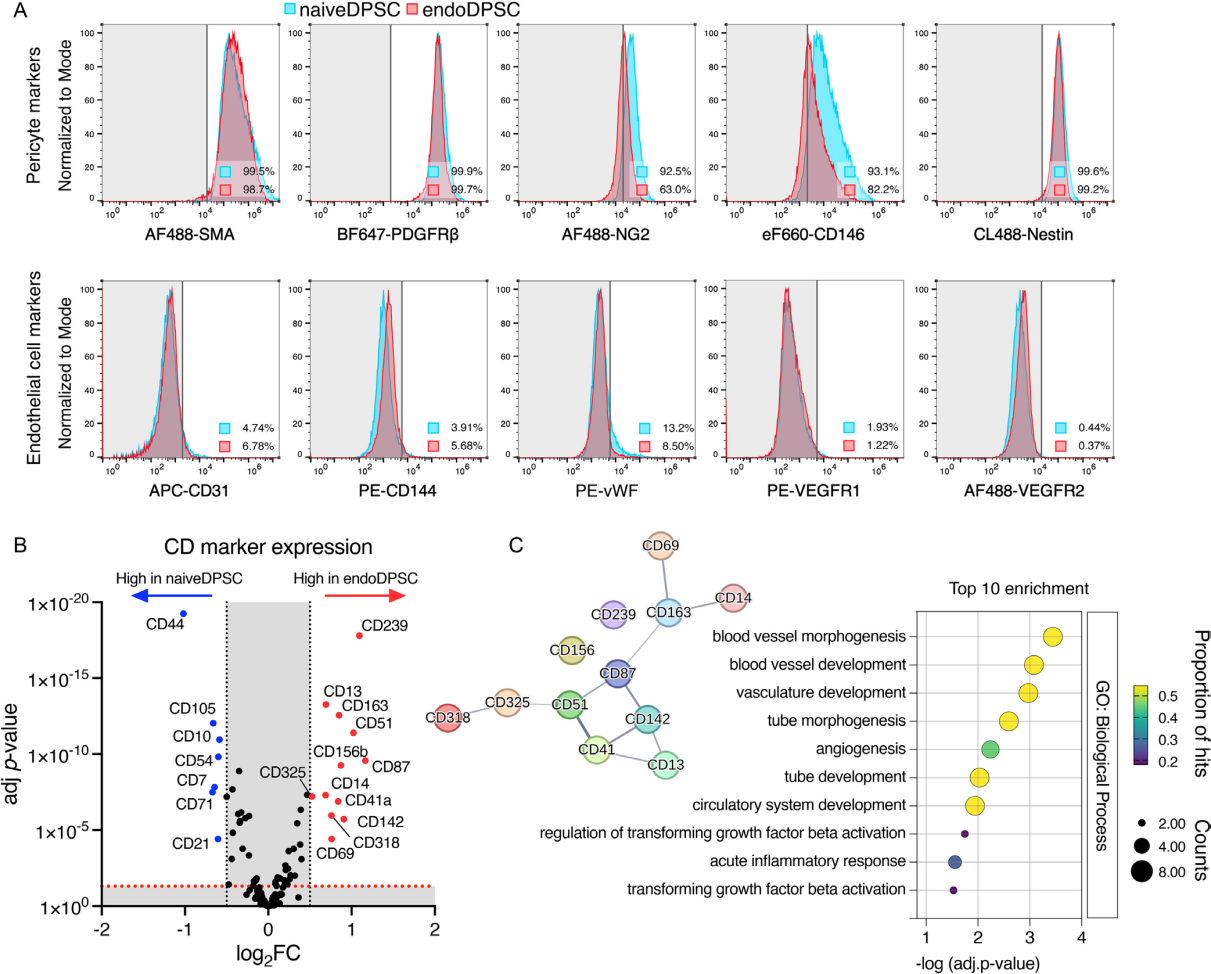
Characterisation of endothelial‐like phenotypes after endothelial stimulation at a protein level. DPSCs were cultured in DMEM (i.e., naiveDPSCs) and EGM2 + VEGF (i.e., endoDPSCs). (A) Flow cytometry analysis targeting pericyte and endothelial cell markers. endoDPSC retained their pericyte‐like phenotypes with marginal reduction of NG2‐ and CD146‐expressin populations, whereas endothelial markers including CD31, CD144, vWF, VEGFR1 and VEGFR2 were found limited in the population. (B) CD marker profiling of endoDPSCs. 12 CD markers were significantly upregulated. (C) The upregulated CD markers were associated with blood vessel morphogenesis and vasculogenesis/angiogenesis predicted by Gene Ontology (GO) assay.

The pro‐angiogenic characteristics and the broader immunophenotype of endoDPSCs were further investigated. Flow cytometry analysis revealed that both naiveDPSCs and endoDPSCs exclusively expressed pericyte markers (α‐SMA, PDGFRβ2, NG2, CD146 and Nestin), while endothelial cell markers (CD31, CD144, vWF, VEGFR1 and VEGFR2) were only marginally expressed, if at all (Figure 4A). Noteworthily, CD31 (+) populations showed a marginal increase from 4.72% in naiveDPSCs to 6.78% in endoDPSCs. The CD marker profiling showed that endoDPSCs significantly increased the expressions of 12 CD markers (Figure 4B, Appendix S1, Table S4). These markers were identified by GO enrichment analysis to be involved in vasculature development and angiogenesis (Figure 4C).

### 3.4 Long‐Term Biobanked DPSCs Demonstrated Pro‐Angiogenic Characteristics in the *ex Ovo* Model

**FIGURE 5:**
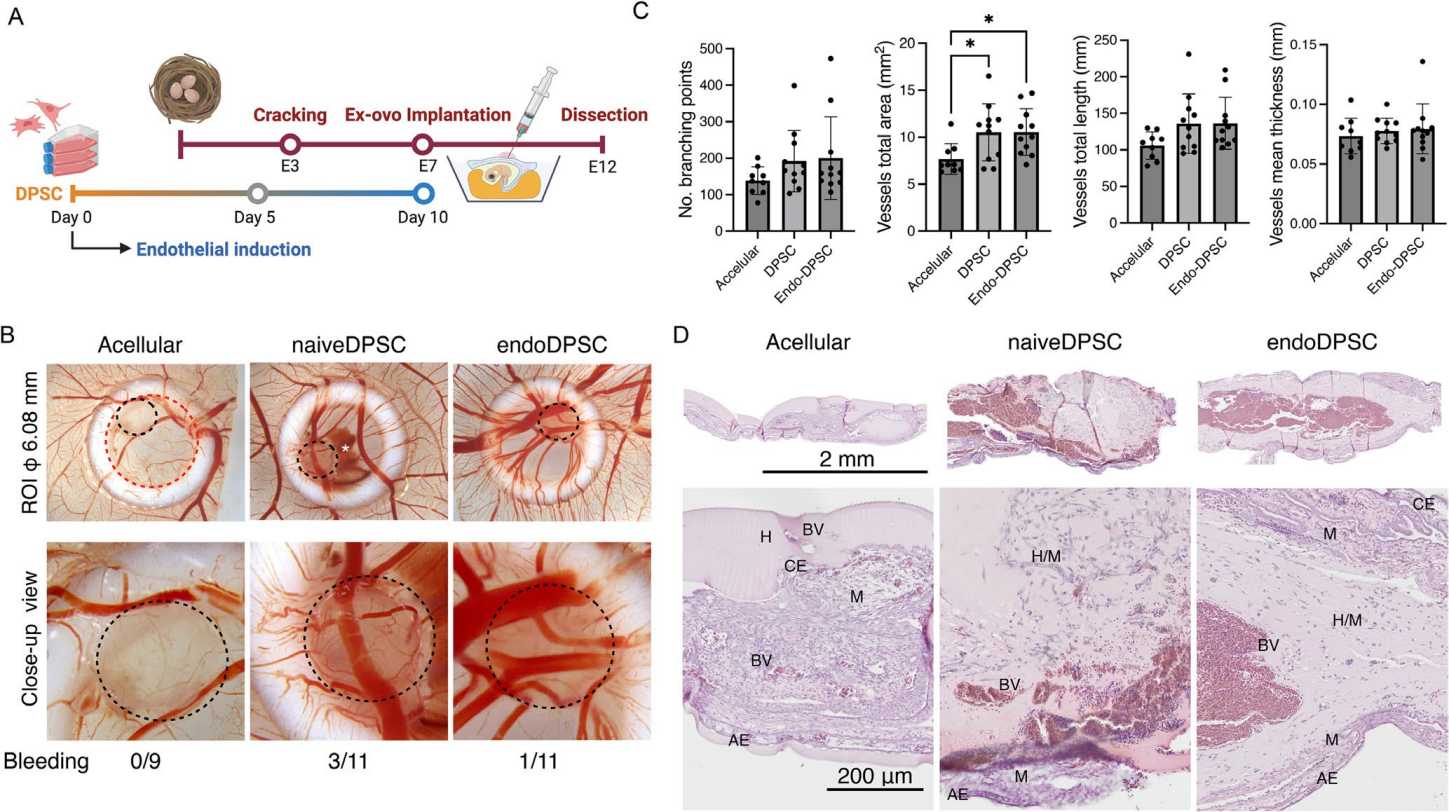
Ex ovo Chorioallantoic membrane (CAM) assay on chicken embryos. (A) Experimental timeline showing the period of endothelial induction followed by implantation. (B) Stereomicroscopic images and (C) blood vessel quantifications of DPSC recipient sites after 5 days of implantation (acellular control *n* = 9, naiveDPSC *n* = 11, endoDPSC *n* = 11). Red circle indicates ROI for vessel quantifications and block circle for implanted samples. (D) haematoxylin and eosin staining of CAM section. BV: Blood vessel; H: Hydrogel; CE: Chorionic ectoderm; M: Mesenchyme; AE: Allantoic endoderm. *p < 0.05.

After 10 days in growth and endothelial conditions, the cells were encapsulated in GFR‐Matrigel and implanted onto the CAM of *ex ovo* chicken embryos (Figure 5A). While the acellular control (i.e., only GFR‐Matrigel) did not promote angiogenesis, both endoDPSCs and naiveDPSCs significantly increased vessel ingrowth, thickening, and branching (Figure 5B,C). Interestingly, when naiveDPSCs were implanted, bleeding around implanted areas was more likely to be observed. H&E staining demonstrated that, while acellular samples were left unintegrated on the surface of the chorionic epithelium, cellular samples in both groups were integrated into the mesoderm with large blood vessel ingrowth, resulting in tissue thickening (Figure 5D).

### 3.5 Biobanked naiveDPSCs Were Capable of Forming Perfusable Vasculatures in Organ‐on‐Chip

**FIGURE 6:**
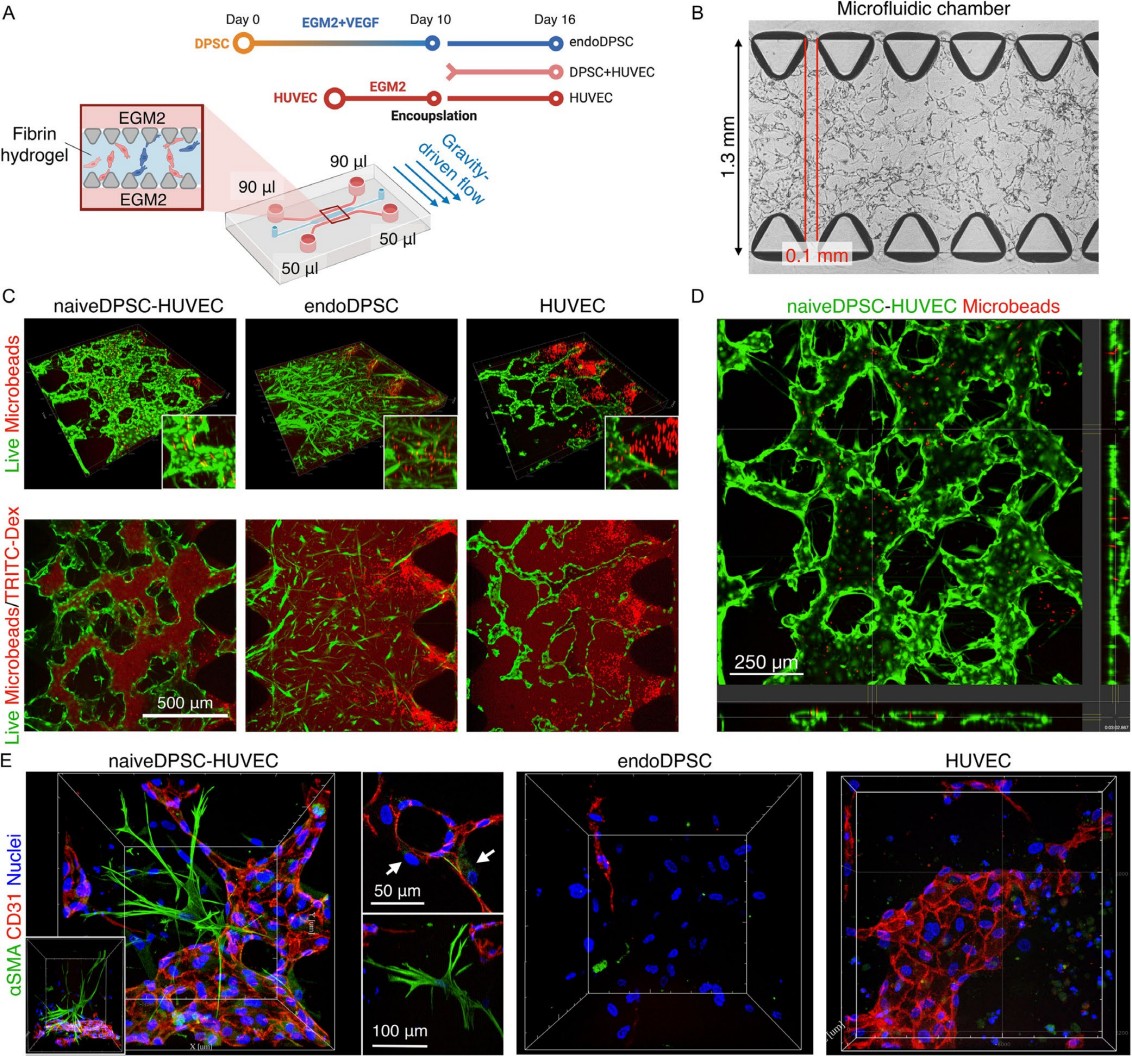
Vascularised dental pulp model using a microfluidic organ‐on‐chip system. (A, B) Experimental timeline and the organ‐on‐chip used in the study. DPSCs were co‐cultured with/without HUVECs under gravity‐driven interstitial‐like fluid flow. (C) Water‐tight perfusable vasculature, visualised by TRITC‐conjugated dextran perfusion, was formed only when naiveDPSCs were co‐cultured with HUVECs. Neither endoDPSCs alone nor HUVECs alone formed functional vasculatures. (D) Microbeads designed to mimic red blood cells were successfully perfused through the engineered vasculature in the organ‐on‐chip system, demonstrating continuous structural integrity and minimal leakage (Movie S1). (E) Immunofluorescence images of αSMA‐expressing and CD31‐expressing cells. When naiveDPSCs and HUVECs were co‐cultured, the DPSCs exhibited myofibroblast‐like and pericyte‐like (arrows) morphology with αSMA expression, supporting vessel formation and stability. endoDPSCs exhibited limited SMA and CD31‐expressing populations. HUVECs exhibited fragmented CD31‐expressing vasculatures.

To understand the mechanism of action behind improved angiogenesis, a microfluidic organ‐on‐chip was employed, combining fibrin hydrogel with/without HUVECs (Figure 6A,B). When cocultured with HUVECs for 6 days, naiveDPSCs formed a watertight lumen structure, which was indicated by no leakage of TRITC‐Dex solution (Figure 6C). In the formed vasculatures, the microbeads mimicking red blood cells were perfused smoothly in the vessels (Figure 6D, Movie S1). Immunofluorescence showed naiveDPSCs spontaneously differentiated into 2 distinct αSMA (+) perivascular populations, demonstrating pericyte‐ and myofibroblast‐like morphologies. These cells were situated around the lumens formed by endothelial cells (Figure 6E). We also tested if endoDPSCs could substitute HUVECs by testing naiveDPSCs co‐cultured with endoDPSCs, but this did not form any visible vasculature (data not shown). This was supported by the monoculture of endoDPSCs, showing that endoDPSCs alone failed to form any visible lumen. However, it is notable that a limited number of CD31 (+) populations was detected. HUVECs alone served as a control. While HUVECs alone generated lumen‐like structures, the generated structure displayed numerous defects, causing leakage and discontinuity despite the dominance of the CD31 (+) population.

### 3.6 Vascularised Tissue Engineering Using Biobanked DPSCs

**FIGURE 7:**
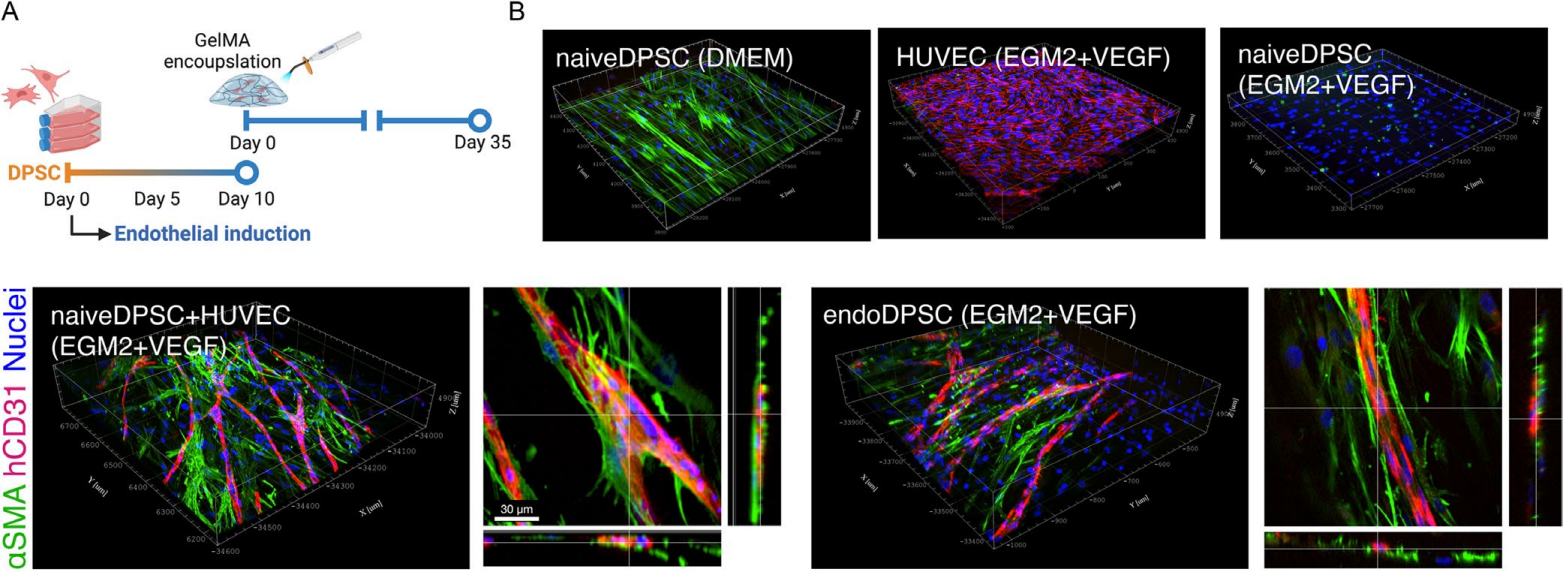
Vascularised tissue engineering using GelMA hydrogels cultured long‐term under endothelial conditions. (A) Experimental design and timeline. DPSCs were cultured under the endothelial inductive condition for 45 days in total. (B) 3D rendering image of immunofluorescence against αSMA and human‐specific CD31. Although both naiveDPSCs + HUVECs co‐culture group and endoDPSCs alone self‐organised vasculatures within the GelMA hydrogel, CD31‐expressing vasculatures were less supported by αSMA‐expressing populations in the endoDPSC group.

The presence of CD31 (+) population, despite a limited number, in the previous experiments led to a hypothesis that functional endothelial populations could be obtained by long‐term exposure to pro‐angiogenic conditions through the selective growth of endothelial subpopulations present in the biobanked DPSCs populations. To validate it, naiveDPSCs and endoDPSCs were encapsulated with/without HUVECs in clinical‐grade GelMA hydrogels and further cultured for 35 days (i.e., in total 45 days culture in the growth or pro‐angiogenic conditions) (Figure 7A).

When naiveDPSCs alone were cultured in growth medium within the hydrogel, strong expression of αSMA was observed. In contrast, when naive DPSCs alone were cultured under endothelial conditions, neither αSMA nor CD31 expression was detected (Figure 7B). As expected, naiveDPSCs cocultured with HUVECs achieved vascularisation of the hydrogels across the construct, proving that biobanked DPSCs retained their pro‐angiogenic characteristics. Strikingly, endoDPSCs alone in pro‐angiogenic conditions also succeeded in neovascularisation with CD31‐expressing endothelial‐like cells supported by αSMA‐expressing cells, observed sporadically yet not consistently, after the prolonged pro‐angiogenic stimuli. This suggests the presence of endothelial‐like populations within the endoDPSCs, which may have either functionally matured (i.e., differentiated) or been selectively expanded during prolonged culture under pro‐angiogenic conditions.

## 4 Discussion

DPSCs are multipotent cells of ectomesenchymal origin, defined by their multipotency and expression of specific surface markers in accordance with ISCT minimum MSC criteria. Clinically, the regenerative potentials of DPSCs have been tested mostly for autologous applications, including dental pulp regeneration, periodontal regeneration, and craniofacial bone regeneration (Mustafa et al. 2025; Yamada et al. 2025). However, MSCs often show significant cellular heterogeneity, a variability further compounded by differences in isolation protocols and culture conditions across laboratories (Dunn et al. 2021). In addition, while cryopreservation is well recognised to preserve the overall cell population and phenotypes, processes including subculturing and long‐term storage have an impact on the population (Cui et al. 2021; Shorokhova et al. 2024; Yu et al. 2010). For example, a scRNAseq study revealed the significant reduction of CD31 (+) subpopulation within DPSCs after monolayer culture (Cui et al. 2021). Long‐term cryopreservation also affects their multipotency and phenotype as a whole due to changes at genomic, epigenetic and transcriptional levels (Shorokhova et al. 2024). At the single cell level, freeze and thaw processes are suggested to influence the composition of MSC subpopulations; however, no study to date has directly compared subpopulation profiles before and after long‐term preservation (Bahsoun et al. 2019).

While most current clinical trials focus on freshly isolated (i.e., non‐cryopreserved) DPSCs, the increasing interest in dental stem cell biobanking and the emerging market landscape are expected to open new opportunities for future regenerative applications using biobanked cells. Therefore, it is of clinical interest if long‐term biobanked DPSCs maintain their ability to promote vascularisation and are suitable for vascularised tissue engineering, which is a critical factor for tissue regeneration.

In the present study, biobanked DPSCs from 4 adolescent donors were characterised for their pro‐angiogenic/vasculogenic characteristics, given angiogenesis is one of the key factors for tissue regeneration. After exposure to the given pro‐angiogenic conditions for 10 days, the cells demonstrated equivalent phenotypes of endothelial‐like DPSCs as previously reported, in terms of morphology, PECAM1 (CD31) upregulation, and tube formation capacity (Bento et al. 2013; Marchionni et al. 2009; Wang et al. 2012). A study on intra‐donor variability of DPSCs demonstrated that over 10% of CD31 positive cells may be present, though not exclusively, in freshly isolated DPSCs, and this population can persist even at passage 7 (Pilbauerova et al. 2021). Additionally, it is known that the degree of root formation at the time of cell isolation impacts the subpopulations, including CD31 (+) endothelium (Tenyi et al. 2023). In the present study, DPSCs were isolated from premolars with closed root apices at the time of extraction. Accordingly, the reduced proportion of CD31(+) cells, especially after long‐term biobanking, was within the expected range. The initial ratio of CD31(+) endothelial subpopulations in these DPSCs may therefore differ from other sources. Commercially available human DPSCs (e.g., from Lonza, Provitro, AcceGen) are typically derived from wisdom teeth of young donors, which may inherently preserve greater subpopulation heterogeneity and more robust functional responses upon stimulation. Indeed, phenotypic differences between primary DPSCs and commercial cell lines have been reported (Macrin et al. 2019). It is worth noting, however, that no functional differences have been reported between upper and lower, or right and left molars in terms of DPSC proliferation, morphology, and multipotency within the same donor, with only two genes found to be differentially expressed at the mRNA level (Faruangsaeng et al. 2022; Pilbauerova et al. 2021). Similarly, rodent DPSCs are commonly isolated from immature molars due to their larger pulp chambers and retained multipotency, or from continuously growing incisors (Ishikawa et al. 2012; Joshi et al. 2022). One study reported robust CD31 protein expression in mouse DPSCs even without endothelial induction (Xu et al. 2025).

In the present study, biobanked DPSCs responded to pro‐angiogenic stimuli by their altered endothelial‐like functionalities and gene expression of endothelial/pro‐angiogenic markers, despite their persistent pericyte‐like phenotypes suggested by flow cytometry. Additionally, the differential CD marker expression in endoDPSCs indicated enrichment in biological processes related to vasculogenesis and angiogenesis. Since the study did not identify which specific subpopulations contributed to the enhanced proangiogenic activity, further investigation using lineage tracing approaches would be beneficial. This is particularly relevant because the ISCT criteria for MSC characterisation do not sufficiently capture the cellular heterogeneity within DPSC populations. Noteworthy is their modulation in NOTCH (+) population after endothelial induction (Figure S1). We observed that the NOTCH1 (+) increased while NOTCH2‐4 (+) cells decreased after endothelial conditioning. In dental pulp, NOTCH expression correlates with cell function and localisation (Krivanek et al. 2020; Mitsiadis et al. 2017; Pagella et al. 2021b). For instance, NOTCH1 is found in vascular‐lining cells, while NOTCH3 is associated with perivascular cells and declines during in vitro differentiation (Mitsiadis et al. 2017; Pagella et al. 2021a). These changes in NOTCH expression in endoDPSCs may reflect a shift in subpopulation composition in response to EGM2 + VEGF stimulation despite further investigation being required. The predicted promotive function on angiogenesis was confirmed by *ex ovo* implantation, proving that DPSCs after long‐term biobanking retain their prominent pro‐angiogenic characteristics, in both naiveDPSC and endoDPSC phenotypes. These angiogenic effects may be attributed to a combination of secretome‐mediated paracrine signalling, partial endothelial transdifferentiation, and the inherent supportive role of pericyte‐like DPSCs in vascular maturation and stability. Given the xenogeneic implantation model, we assume that the observed pro‐angiogenic effects were primarily mediated by the DPSC secretome. Indeed, proteomic analysis revealed that DPSCs secrete a broad array of factors involved in vasculogenesis and angiogenesis (Figure S2). Nevertheless, previous studies in rodent models have indicated that a subpopulation of transplanted DPSCs may also contribute directly to neovascularisation by differentiating into CD31(+) endothelial‐like cells (Marchionni et al. 2009; Wang et al. 2012; Zhang et al. 2021).

Building on the confirmed pro‐angiogenic properties, we finally assessed whether biobanked DPSCs could be applied to vascularised tissue engineering. In an organ‐on‐chip model cultured for up to one week, naive DPSCs combined with HUVECs successfully formed perfusable, water‐tight vascularised tissues. Notably, naive DPSCs spontaneously differentiated into distinct perivascular cell types, exhibiting both small, pericyte‐like and large, myofibroblast‐like morphologies, and established direct physical contact with HUVECs. Noteworthily, while endoDPSCs alone in the chip did not form any vasculature, a handful number of CD31 (+) was observed. This led to a hypothesis that long‐term endothelial cell culture conditions may selectively propagate the endothelial progenitors in the DPSC population. Strikingly, we successfully engineered vascularised tissue using DPSCs alone in a clinical‐grade GelMA hydrogel after 45 days of endothelial conditioning. The resulting tissue exhibited both CD31 (+) endothelial‐like and αSMA (+) perivascular cells, and its structural organisation was comparable to that formed by naive DPSCs co‐cultured with HUVECs. Although the vasculature formed was sporadic, likely due to the limited presence of endothelial‐like populations, these findings highlight the intrinsic potential of DPSCs to contribute to neovascularisation. Further optimisation of endothelial induction protocols, including medium composition, additive selection, and growth factor concentrations, may enhance the efficiency and consistency of vascularised tissue formation.

DPSC heterogeneity plays a critical role in angiogenesis. A side population of DPSCs, which cannot be distinguished by surface markers but is identified by its ability to efflux DNA‐binding dyes, has been recognised as a superior multipotent subset with enhanced endothelial potential upon induction (Wang et al. 2012). In contrast, a previous study showed that the CD31 (−) subpopulation in DPSCs promoted angiogenesis more robustly through rich angiogenic factor secretion (Ishizaka et al. 2013). These findings suggest that heterogeneous DPSC subsets may interact synergistically to support vascularisation. The present study demonstrates that long‐term biobanked DPSCs retain both substantial pro‐angiogenic potential and inherent cellular heterogeneity. To the best of our knowledge, this is the first report to show that biobanked human DPSCs, without the need for additional cell types or genetic modification, can independently support the formation of vascularised tissue in vitro through a tissue engineering approach. The mechanism underlying successful vascularised tissue formation appears to be multifactorial. It likely involves a synergistic interplay between: (1) pro‐angiogenic phenotypes induced by endothelial‐inductive culture conditions; (2) intrinsic pericyte‐like characteristics of DPSCs, which are functionally enhanced by interactions with endothelial cells; (3) a secretome enriched in angiogenic mediators; and (4) the selective expansion of endothelial progenitor‐like subpopulations within the heterogeneous DPSC pool. These combined properties, rather than any single mechanism alone, are critical in promoting neovascularisation.

The isolation of DPSCs is uniquely feasible at the time of routine dental procedures, such as tooth extraction (Yamada et al. 2025). Long‐term biobanking of dental stem cells is therefore emerging as a practical strategy to meet future therapeutic demands, particularly as cell‐based therapies show increasing promise in dentistry. As a result, a growing number of academic institutions and commercial providers now offer biobanking services for dental‐derived stem cells intended for future clinical use. This “just‐in‐case” biobanking strategy significantly enhances the accessibility and clinical practicality of DPSCs as a personalised, off‐the‐shelf resource for regenerative interventions. In contrast, the isolation of autologous dental stem cells for immediate clinical use remains uncommon and often requires cryopreservation steps. Despite its apparent simplicity, the process involves complex biophysical and biochemical dynamics that may influence cell viability, proliferation, differentiation capacity, and genomic stability depending on protocols (Wang et al. 2023). While evidence on the long‐term biobanking of DPSCs remains limited, existing studies suggest that their functionality and phenotype can be influenced by the specific cryopreservation protocols used. Notably, in DPSCs, the expression level of miR‐218 has been identified as a potential determinant of post‐thaw survival following long‐term storage up to 10 years (Lott et al. 2022). However, data beyond the 10‐year storage are currently unavailable for dental stem cells. In contrast, studies on BMSCs have shown that cryopreservation exceeding 10 years can result in a significant decline in cell viability, post‐thaw proliferation, and osteogenic differentiation potency despite notable donor variability (Sugimoto et al. 2021). A similar trend has been reported in ASCs (Shaik et al. 2018).

Notably, all of these studies, including the present work, employed cryopreservation media containing xenogeneic components such as FBS. While the use of FBS remains common in preclinical research and was previously widespread in early clinical studies, regulatory authorities including the European Medicines Agency (EMA; EMEA/CHMP/410869/2006) and the US Food and Drug Administration (FDA; FDA‐2024‐D‐1244, draft) now strongly recommend avoiding xenogeneic products in clinical cell manufacturing. This regulatory shift is reflected in the limited number of studies that have explored the use of cryopreserved dental stem cells with xeno‐free cryopreservation media, highlighting a critical gap in the translational pipeline for clinical‐grade biobanking (Yamada et al. 2025). Additionally, while standard cryopreservation protocols for both research and clinical use exclusively rely on 5%–10% DMSO as cryoprotectant (Wang and Li 2024), DMSO, even at low concentrations (e.g., 0.1%), is known to significantly alter cellular genetic, epigenetic and transcriptomic profiles (Redaelli et al. 2012; Verheijen et al. 2019). As safer alternatives, emerging strategies are being explored to include sugar‐based cryoprotectants (Izaguirre‐Pérez et al. 2024; Tripathy et al. 2022), polyvinyl alcohol (PVA) (Wang et al. 2011), polyampholytes (Matsumura and Hyon 2009), and various nano‐sized materials as non‐toxic cryoprotectant delivery systems (Huang et al. 2021). Moreover, critical parameters such as cooling rates, the type of medium and additives, and rewarming methods are also likely to influence post‐thaw outcomes. Despite these advances, current evidence on long‐term cryopreservation remains limited and lacks clinical applicability. Further investigation is urgently needed to assess the long‐term effects of clinical‐grade cryopreservation protocols on the viability, safety and functionality of stem cell products intended for therapeutic use.

Lastly, we explored vascularisation as a key aspect of tissue regeneration and found that DPSCs cryopreserved for over seven years retained a robust proangiogenic effect in both in vitro and *ex ovo* models. However, this study did not directly assess the extent to which this effect was preserved compared to freshly isolated DPSCs. It is likely that phenotypic changes and shifts in subpopulations during long‐term storage contribute to functional alterations. Ideally, donor‐matched comparison, tested both fresh and after long‐term storage, would provide the most reliable data. However, this approach is technically challenging due to the long timeframes involved and the evolving nature of protocols, reagents and analytical standards, which make direct comparisons difficult. As a mitigation strategy, comparisons between freshly isolated and biobanked cells from different donors could be considered. Yet, such studies must account for donor‐to‐donor variability, which would require a sufficiently large cohort to enable statistically and biologically meaningful conclusions. This study provides evidence at the given protocols that long‐term biobanked DPSCs retain proangiogenic potential, while leaving open important questions regarding the comparison to fresh cells and the mechanisms underlying any functional changes.

## 5 Conclusion

This study demonstrates, for the first time, that long‐term biobanked human DPSCs retain their pro‐angiogenic capacity and can generate vascularised tissues in vitro. Biobanked DPSCs exhibited robust responses to pro‐angiogenic stimuli, with enhanced expression of angiogenic and endothelial‐related genes and functional tube formation. Despite the altered gene expression, their perivascular phenotypes remained predominant in vitro. *Ex ovo* and organ‐on‐chip models confirmed their angiogenic potential, suggesting a dual role through both paracrine secretion and pericyte‐like support behind promoted angiogenesis. Strikingly, prolonged 3D culture in clinical‐grade GelMA hydrogels showed that DPSCs alone could generate CD31 (+) vasculatures supported by SMA (+) side population after 45 days of endothelial conditioning. These findings underscore the translational promise of biobanked DPSCs for vascularised tissue engineering, supporting their clinical utility in regenerative dentistry and beyond.

## Author Contributions

S. Yamada contributed to funding acquisition, conception, design, data acquisition, analysis and interpretation, drafted and critically revised the manuscript; K. Holomkova, Å. Johansen, M.J. Kadousaraei, N. Al‐Sharabi and F. Torelli contributed data acquisition, analysis and critically revised the manuscript; P. Pagella, A.A. Volponi and H. Egusa contributed design, interpretation and critically revised the manuscript. I. Fristad and K. Mustafa contributed funding acquisition, conception and revised the manuscript. All authors gave final approval and agreed to be accountable for all aspects of the work.

## Conflicts of Interest

The authors declare no conflicts of interest.

## Supporting information


**Figure S1:** NOTCH expression in naiveDPSCs and endoDPSCs. Flow cytometry analysis of NOTCH1‐4. After endothelial induction, DPSCs notably altered their NOTCH expression, with NOTCH1‐expressing population increased and NOTCH2‐4 decreased.
**Figure S2:** Mass‐spectroscopy‐based proteomics analysis of secretome from DPSCs. Among 2442 proteins identified, 192 proteins were predicted to be involved in vasculature development, 185 were in blood vessel development, 145 were in angiogenesis (left). Gene Ontology (GO) analysis of identified proteins (right). Methodology of the proteomics is described previously (Yamada et al. 2024).

**Movie S1:** Time‐lapse recording of fully perfusable vasculature formation in a microfluidic organ‐on‐chip. Co‐culture of DPSCs and HUVECs produced water‐tight, functional vessel networks. Images were acquired by Dragonfly confocal microscope over 3 min at 5‐s intervals and compiled at 10 fps. Green: cells stained with Calcein AM; Red: 4‐μm microbeads.

## Data Availability

The data that support the findings of this study are available from the corresponding author upon reasonable request.

## References

[iej70036-bib-0001] Bahsoun, S. , K. Coopman , and E. C. Akam . 2019. “The Impact of Cryopreservation on Bone Marrow‐Derived Mesenchymal Stem Cells: A Systematic Review.” Journal of Translational Medicine 17, no. 1: 397. 31783866 10.1186/s12967-019-02136-7 PMC6883667

[iej70036-bib-0002] Bento, L. W. , Z. Zhang , A. Imai , et al. 2013. “Endothelial Differentiation of Shed Requires mek1/Erk Signaling.” Journal of Dental Research 92, no. 1: 51–57. 23114032 10.1177/0022034512466263 PMC3521451

[iej70036-bib-0003] Bergamo, M. T. , Z. Zhang , T. M. Oliveira , and J. E. Nör . 2021. “Vegfr1 Primes a Unique Cohort of Dental Pulp Stem Cells for Vasculogenic Differentiation.” European Cells and Materials 41: 332–344. 33724439 10.22203/eCM.v041a21 PMC8561749

[iej70036-bib-0004] Cui, Y. , W. Ji , Y. Gao , Y. Xiao , H. Liu , and Z. Chen . 2021. “Single‐Cell Characterization of Monolayer Cultured Human Dental Pulp Stem Cells With Enhanced Differentiation Capacity.” International Journal of Oral Science 13, no. 1: 44. 34911932 10.1038/s41368-021-00140-6 PMC8674359

[iej70036-bib-0005] Dominici, M. , K. Le Blanc , I. Mueller , et al. 2006. “Minimal Criteria for Defining Multipotent Mesenchymal Stromal Cells. The International Society for Cellular Therapy Position Statement.” Cytotherapy 8, no. 4: 315–317. 16923606 10.1080/14653240600855905

[iej70036-bib-0006] Dunn, C. M. , S. Kameishi , D. W. Grainger , and T. Okano . 2021. “Strategies to Address Mesenchymal Stem/Stromal Cell Heterogeneity in Immunomodulatory Profiles to Improve Cell‐Based Therapies.” Acta Biomaterialia 133: 114–125. 33857693 10.1016/j.actbio.2021.03.069

[iej70036-bib-0007] Egusa, H. , W. Sonoyama , M. Nishimura , I. Atsuta , and K. Akiyama . 2012. “Stem Cells in Dentistry–Part i: Stem Cell Sources.” Journal of Prosthodontic Research 56, no. 3: 151–165. 22796367 10.1016/j.jpor.2012.06.001

[iej70036-bib-0008] Faruangsaeng, T. , S. Thaweesapphitak , C. Khamwachirapitak , T. Porntaveetus , and V. Shotelersuk . 2022. “Comparative Transcriptome Profiles of Human Dental Pulp Stem Cells From Maxillary and Mandibular Teeth.” Scientific Reports 12, no. 1: 8860. 35614192 10.1038/s41598-022-12867-1 PMC9133121

[iej70036-bib-0009] Ganapathy, A. , K. Narayanan , Y. Chen , C. Villani , and A. George . 2024. “Dentin Matrix Protein 1 and Huvec‐Ecm Scaffold Promote the Differentiation of Human Dental Pulp Stem Cells Into Endothelial Lineage: Implications in Regenerative Medicine.” Frontiers in Physiology 15: 1429247. 39040080 10.3389/fphys.2024.1429247 PMC11260688

[iej70036-bib-0010] Huang, J. , J. Guo , L. Zhou , et al. 2021. “Advanced Nanomaterials‐Assisted Cell Cryopreservation: A Mini Review.” ACS Applied Bio Materials 4, no. 4: 2996–3014. 10.1021/acsabm.1c00105 35014388

[iej70036-bib-0011] Ishikawa, Y. , H. Ida‐Yonemochi , K. Nakakura‐Ohshima , and H. Ohshima . 2012. “The Relationship Between Cell Proliferation and Differentiation and Mapping of Putative Dental Pulp Stem/Progenitor Cells During Mouse Molar Development by Chasing Brdu‐Labeling.” Cell and Tissue Research 348, no. 1: 95–107. 22370596 10.1007/s00441-012-1347-2

[iej70036-bib-0012] Ishizaka, R. , Y. Hayashi , K. Iohara , et al. 2013. “Stimulation of Angiogenesis, Neurogenesis and Regeneration by Side Population Cells From Dental Pulp.” Biomaterials 34, no. 8: 1888–1897. 23245334 10.1016/j.biomaterials.2012.10.045

[iej70036-bib-0013] Itoh, Y. , J. I. Sasaki , M. Hashimoto , C. Katata , M. Hayashi , and S. Imazato . 2018. “Pulp Regeneration by 3‐Dimensional Dental Pulp Stem Cell Constructs.” Journal of Dental Research 97, no. 10: 1137–1143. 29702010 10.1177/0022034518772260

[iej70036-bib-0014] Izaguirre‐Pérez, N. , G. Ligero , P. A. Aguilar‐Solana , et al. 2024. “Trehalose Cryopreservation of Human Mesenchymal Stem Cells From Cord Tissue.” Biopreservation and Biobanking 23, no. 4: 374–382. 39723442 10.1089/bio.2024.0025

[iej70036-bib-0015] Janebodin, K. , Y. Zeng , W. Buranaphatthana , N. Ieronimakis , and M. Reyes . 2013. “Vegfr2‐Dependent Angiogenic Capacity of Pericyte‐Like Dental Pulp Stem Cells.” Journal of Dental Research 92, no. 6: 524–531. 23609159 10.1177/0022034513485599

[iej70036-bib-0016] Joshi, P. , A. Vijaykumar , B. Enkhmandakh , D. G. Shin , M. Mina , and D. Bayarsaihan . 2022. “The Chromatin Accessibility Landscape in the Dental Pulp of Mouse Molars and Incisors.” Acta Biochimica Polonica 69, no. 1: 131–138. 35226446 10.18388/abp.2020_5771

[iej70036-bib-0017] Katata, C. , J. I. Sasaki , A. Li , et al. 2021. “Fabrication of Vascularized Dpsc Constructs for Efficient Pulp Regeneration.” Journal of Dental Research 100, no. 12: 1351–1358. 33913364 10.1177/00220345211007427 PMC9290113

[iej70036-bib-0018] Krivanek, J. , R. A. Soldatov , M. E. Kastriti , et al. 2020. “Dental Cell Type Atlas Reveals Stem and Differentiated Cell Types in Mouse and Human Teeth.” Nature Communications 11, no. 1: 4816. 10.1038/s41467-020-18512-7 PMC7511944 32968047

[iej70036-bib-0019] Li, A. , J. I. Sasaki , T. Inubushi , et al. 2023. “Role of Heparan Sulfate in Vasculogenesis of Dental Pulp Stem Cells.” Journal of Dental Research 102, no. 2: 207–216. 36281071 10.1177/00220345221130682 PMC10767696

[iej70036-bib-0020] Liu, Y. , L. Gan , D. X. Cui , et al. 2021. “Epigenetic Regulation of Dental Pulp Stem Cells and Its Potential in Regenerative Endodontics.” World Journal of Stem Cells 13, no. 11: 1647–1666. 34909116 10.4252/wjsc.v13.i11.1647 PMC8641018

[iej70036-bib-0021] Lott, K. , P. Collier , M. Ringor , et al. 2022. “Assessment and Analysis of Dental Pulp Stem Cells (Dpscs) Biomarkers and Viability Following Cryopreser‐Vation Reveals Novel Association With Mir‐218 Expression.” EC Dental Science 21: 115–128.

[iej70036-bib-0022] Ma, X. , B. Zhao , C. Wang , et al. 2024. “Anxa1 Enhances the Proangiogenic Potential of Human Dental Pulp Stem Cells.” Stem Cells International 2024, no. 1: 7045341. 39478978 10.1155/2024/7045341 PMC11524703

[iej70036-bib-0023] Macrin, D. , A. Alghadeer , Y. T. Zhao , et al. 2019. “Metabolism as an Early Predictor of Dpscs Aging.” Scientific Reports 9, no. 1: 2195. 30778087 10.1038/s41598-018-37489-4 PMC6379364

[iej70036-bib-0024] Marchionni, C. , L. Bonsi , F. Alviano , et al. 2009. “Angiogenic Potential of Human Dental Pulp Stromal (Stem) Cells.” International Journal of Immunopathology and Pharmacology 22, no. 3: 699–706. 19822086 10.1177/039463200902200315

[iej70036-bib-0025] Matsumura, K. , and S. H. Hyon . 2009. “Polyampholytes as Low Toxic Efficient Cryoprotective Agents With Antifreeze Protein Properties.” Biomaterials 30, no. 27: 4842–4849. 19515417 10.1016/j.biomaterials.2009.05.025

[iej70036-bib-0026] Mitsiadis, T. , J. Catón , P. Pagella , G. Orsini , and L. Jimenez‐Rojo . 2017. “Monitoring Notch Signaling‐Associated Activation of Stem Cell Niches Within Injured Dental Pulp.” Frontiers in Physiology 8: 372. 28611689 10.3389/fphys.2017.00372 PMC5447770

[iej70036-bib-0027] Mustafa, K. , S. Yamada , N. Sanchez , M. Mayol , C. Gjerde , and M. Sanz . 2025. “Cell Therapy for Periodontal, Soft‐Tissue, and Craniofacial Regeneration.” Journal of Periodontal Research: 1–26. 40616236 10.1111/jre.70011

[iej70036-bib-0028] Nagendrababu, V. , P. E. Murray , R. Ordinola‐Zapata , et al. 2021. “Prile 2021 Guidelines for Reporting Laboratory Studies in Endodontology: A Consensus‐Based Development.” International Endodontic Journal 54, no. 9: 1482–1490. 33938010 10.1111/iej.13542

[iej70036-bib-0029] Pagella, P. , L. de Vargas Roditi , B. Stadlinger , A. E. Moor , and T. A. Mitsiadis . 2021a. “Notch Signaling in the Dynamics of Perivascular Stem Cells and Their Niches.” Stem Cells Translational Medicine 10, no. 10: 1433–1445. 34227747 10.1002/sctm.21-0086 PMC8459638

[iej70036-bib-0030] Pagella, P. , L. de Vargas Roditi , B. Stadlinger , A. E. Moor , and T. A. Mitsiadis . 2021b. “A Single‐Cell Atlas of Human Teeth.” iScience 24, no. 5: 102405. 33997688 10.1016/j.isci.2021.102405 PMC8099559

[iej70036-bib-0031] Pilbauerova, N. , J. Schmidt , T. Soukup , J. Duska , and J. Suchanek . 2021. “Intra‐Individual Variability of Human Dental Pulp Stem Cell Features Isolated From the Same Donor.” International Journal of Molecular Sciences 22, no. 24: 13515. 34948330 10.3390/ijms222413515 PMC8709021

[iej70036-bib-0032] Redaelli, S. , A. Bentivegna , D. Foudah , et al. 2012. “From Cytogenomic to Epigenomic Profiles: Monitoring the Biologic Behavior of In Vitro Cultured Human Bone Marrow Mesenchymal Stem Cells.” Stem Cell Research & Therapy 3, no. 6: 47. 23168092 10.1186/scrt138 PMC3580477

[iej70036-bib-0033] Sasaki, J. I. , Z. Zhang , M. Oh , et al. 2020. “Ve‐Cadherin and Anastomosis of Blood Vessels Formed by Dental Stem Cells.” Journal of Dental Research 99, no. 4: 437–445. 32028818 10.1177/0022034520902458 PMC7088203

[iej70036-bib-0034] Shaik, S. , X. Wu , J. Gimble , and R. Devireddy . 2018. “Effects of Decade Long Freezing Storage on Adipose Derived Stem Cells Functionality.” Scientific Reports 8, no. 1: 8162. 29802353 10.1038/s41598-018-26546-7 PMC5970158

[iej70036-bib-0035] Shorokhova, M. , N. Pugovkina , V. Zemelko , O. Lyublinskaya , and T. Grinchuk . 2024. “Long‐Term Cryopreservation May Cause Genomic Instability and the Premature Senescence of Cells.” International Journal of Molecular Sciences 25, no. 3: 1467. 38338745 10.3390/ijms25031467 PMC10855830

[iej70036-bib-0036] Sugimoto, Y. , Y. Yamazaki , K. Moriyama , et al. 2021. “Differentiation and Proliferation Potencies of Human Bone Tissue‐Derived Mesenchymal Stromal Cells (Hbt‐Mscs) After Long‐Term Cryopreservation ‐Comparison Among Cells Stored for 1, 5, 10, 15, and 20 Years.” Regenerative Therapy 18: 363–371. 34632009 10.1016/j.reth.2020.01.006 PMC8473671

[iej70036-bib-0037] Tenyi, A. , A. Milutinović , and L. Nemeth . 2023. “Expression of cd31, cd34, and Smooth Muscle Actin (Sma) in Endothelial Cells of Dental Pulp Vessels.” Biomolecules & Biomedicine 24, no. 4: 821–826. 38153414 10.17305/bb.2023.9988 PMC11293224

[iej70036-bib-0038] Thaler, R. , S. Spitzer , H. Karlic , K. Klaushofer , and F. Varga . 2012. “Dmso Is a Strong Inducer of DNA Hydroxymethylation in Pre‐Osteoblastic mc3t3‐e1 Cells.” Epigenetics 7, no. 6: 635–651. 22507896 10.4161/epi.20163 PMC3398991

[iej70036-bib-0039] Tripathy, S. , S. Singh , and S. K. Das . 2022. “Chapter 19 ‐ Cryopreservation of Mesenchymal Stem Cells (Mscs) Derived From Bone Marrow With Carbohydrate Additive Sucrose and Dimethyl Sulfoxide (Dmso).” In Contemporary Medical Biotechnology Research for Human Health, edited by S. Joshi , S. Mukherjee , and M. Nag , 177–186. Academic Press.

[iej70036-bib-0040] Verheijen, M. , M. Lienhard , Y. Schrooders , et al. 2019. “Dmso Induces Drastic Changes in Human Cellular Processes and Epigenetic Landscape In Vitro.” Scientific Reports 9, no. 1: 4641. 30874586 10.1038/s41598-019-40660-0 PMC6420634

[iej70036-bib-0041] Wang, H. Y. , Z. R. Lun , and S. S. Lu . 2011. “Cryopreservation of Umbilical Cord Blood‐Derived Mesenchymal Stem Cells Without Dimethyl Sulfoxide.” Cryo Letters 32, no. 1: 81–88. 21468457

[iej70036-bib-0042] Wang, J. , and R. Li . 2024. “Effects, Methods and Limits of the Cryopreservation on Mesenchymal Stem Cells.” Stem Cell Research & Therapy 15, no. 1: 337. 39343920 10.1186/s13287-024-03954-3 PMC11441116

[iej70036-bib-0043] Wang, J. , X. Wei , J. Ling , Y. Huang , Q. Gong , and Y. Huo . 2012. “Identification and Characterization of Side Population Cells From Adult Human Dental Pulp After Ischemic Culture.” Journal of Endodontics 38, no. 11: 1489–1497. 23063223 10.1016/j.joen.2012.08.004

[iej70036-bib-0044] Wang, X. , E. Wang , and G. Zhao . 2023. “Advanced Cryopreservation Engineering Strategies: The Critical Step to Utilize Stem Cell Products.” Cell Regeneration 12, no. 1: 28. 37528321 10.1186/s13619-023-00173-8 PMC10393932

[iej70036-bib-0045] Xu, X. , J. Fu , G. Yang , Z. Chen , S. Chen , and G. Yuan . 2025. “Dentin Sialoprotein Promotes Endothelial Differentiation of Dental Pulp Stem Cells Through Dsp(aa34‐50)‐Endoglin‐akt1 Axis.” Journal of Biological Chemistry 301, no. 4: 108380. 40049415 10.1016/j.jbc.2025.108380 PMC11997338

[iej70036-bib-0046] Yamada, S. , N. Al‐Sharabi , F. Torelli , et al. 2024. “Harnessing the Antioxidative Potential of Dental Pulp Stem Cell‐Conditioned Medium in Photopolymerized Gelma Hydrogels.” Biomaterials Research 28: 0084. 39290361 10.34133/bmr.0084 PMC11406670

[iej70036-bib-0047] Yamada, S. , C. Malkmus , E. Aasebø , K. Mustafa , H. Egusa , and A. A. Volponi . 2025. “Production and Biobanking of Dental Stem Cells for Clinical Applications in Regenerative Dentistry: Current Practices and Future Perspectives ‐ a Narrative Review.” Journal of Dentistry 161: 105934. 40578781 10.1016/j.jdent.2025.105934

[iej70036-bib-0048] Yu, J. , H. He , C. Tang , et al. 2010. “Differentiation Potential of Stro‐1+ Dental Pulp Stem Cells Changes During Cell Passaging.” BMC Cell Biology 11, no. 1: 32. 20459680 10.1186/1471-2121-11-32 PMC2877667

[iej70036-bib-0049] Zhang, Z. , M. Oh , J.‐I. Sasaki , and J. E. Nör . 2021. “Inverse and Reciprocal Regulation of p53/p21 and Bmi‐1 Modulates Vasculogenic Differentiation of Dental Pulp Stem Cells.” Cell Death & Disease 12, no. 7: 644. 34168122 10.1038/s41419-021-03925-z PMC8225874

