# Supplementary material for: Long‐Term Biobanked Dental Pulp Stem Cells Retain Angiogenic Potential for Vascularised Tissue Engineering—Laboratory Investigation
Source: Int Endod J. 2025 Sep 23;59(1):105–18. doi: 10.1111/iej.70036 (PMC12701750; doi:10.1111/iej.70036)
Supplement: Supplementary file 1 — Figure S1: NOTCH expression in naiveDPSCs and endoDPSCs. Flow cytometry analysis of NOTCH1‐4. After endothelial induction, DPSCs notably altered their NOTCH expression, with NOTCH1‐expressing population increased and NOTCH2‐4 decreased. Figure S2: Mass‐spectroscopy‐based proteomics analysis of secretome from DPSCs. Among 2442 proteins identified, 192 proteins were predicted to be involved in vasculature development, 185 were in blood vessel development, 145 were in angiogenesis (left). Gene Ontology (GO) analysis of identified proteins (right). Methodology of the proteomics is described previously (Yamada et al. 2024). [file IEJ-59-105-s001.docx]

**Long-Term Biobanked Dental Pulp Stem Cells Retain Angiogenic Potential for Vascularized Tissue Engineering - Laboratory Investigation**

Shuntaro Yamada^1,2^*, Katerina Holomkova^1,3,4＃^, Åshild Johansen^1＃^, Masoumeh Jahani Kadousaraei^1^, Niyaz Al-Sharabi^1^, Francesco Torelli^1^, Pierfrancesco Pagella^5^, Ana Angelova Volponi^2^, Hiroshi Egusa^6,7^, Inge Fristad^1^, Kamal Mustafa^1^*

1. Center of Translational Oral Research - Tissue Engineering, Department of Clinical Dentistry, University of Bergen, Bergen, Norway

2. Centre for Craniofacial & Regenerative Biology, Faculty of Dentistry, Oral & Craniofacial Sciences, King’s College London, London, UK

3. Department of Histology and Embryology, Faculty of Medicine, Masaryk University, Brno, Czechia

4. Institute of Animal Physiology and Genetics, Czech Academy of Sciences, Brno, Czechia

5. Laboratory of Molecular Materials, Division of Biophysics and Bioengineering, Department of Physics, Chemistry and Biology (IFM), Linköping University, Linköping, Sweden

6. Center for Advanced Stem Cell and Regenerative Research, Tohoku University Graduate School of Dentistry, Miyagi, Japan

7. Division of Molecular & Regenerative Prosthodontics, Tohoku University Graduate School of Dentistry, Miyagi, Japan

^＃^The authors equally contributed to the work

* Corresponding author

Shuntaro Yamada: [Shuntaro.yamada@uib.no](mailto:Shuntaro.yamada@uib.no), Årstadveien 19, 5009 Bergen
Kamal Mustafa: [kamal.mustafa@uib.no](mailto:kamal.mustafa@uib.no), Årstadveien 19, 5009 Bergen


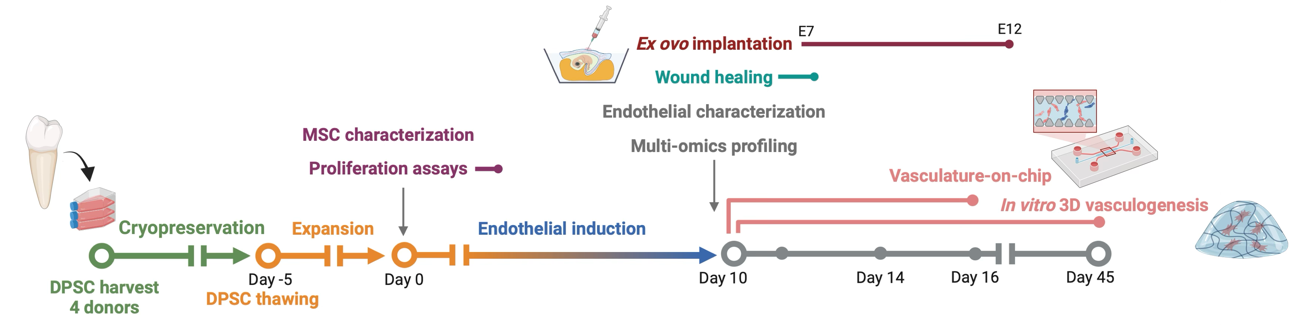


**Graphical abstract of study design**

1. **Appendix Materials & Methods:**
   1. **MSC characterization and multilineage differentiation**

DPSCs from 4 donors were cultured in the growth medium consisting DMEM with 10% FBS and pooled at passage 4 for MSC characterization. For evaluation of their multipotency, DPSCs were induced into osteogenic, chondrogenic, adipogenic, and neurogenic lineages.

For osteogenic differentiation, DPSCs were cultured under osteogenic medium consisting of Dulbecco’s modified Eagle’s medium (DMEM; 10566016, Gibco, USA) with 10% fetal bovine serum (FBS; 10270-106; Gibco, USA) and 1% penicillin/streptomycin (SV30010; HyClone, USA), supplemented with 200 nM dexamethasone, 10 mM glycerophosphate and 0.05 mM ascorbic acid, for 14 days at 37°C in 5% CO_2_ humidified atmosphere. The cells were then fixed in 4% PFA for 40 min and washed three times with Milli-Q^®^ water. The samples were incubated with 0.1% Alizarin Red S for 20 min followed by 3 time-wash with Milli-Q® water.

For adipogenic differentiation, DPSCs were cultured in MesenCult™ Adipogenic Differentiation Medium (05412, STEMCELL Technologies, USA) for 14 days. Subsequently, the cells were fixed in 4% PFA. Fat droplets were stained using BODIPY (493/503) dye (D3922, Invitrogen, USA). The nuclei and filamentous actin were counterstained by Alexa Fluor 488 Phalloidin (A12379, Invitrogen, USA) and Hoechst 33342 (B2261, Sigma-Aldrich, USA). The cells were visualized using a confocal microscope (Andor Dragonfly 505, Oxford Instruments, UK).

For chondrogenic differentiation, the cells were cultured as three-dimensional (3D) pellets in chondrogenic differentiation medium (CCM000/CCM020, R&D Systems, USA), following the manufacturer’s instructions. After 21 days of incubation, the pellets were fixed, embedded in paraffin, and sectioned into 5 μm-thick slices. The samples were stained with 1% Alcian Blue (pH 2.5; A5268, Sigma-Aldrich, USA) dissolved in acetic acid for 30 min at RT. The tissue was then counterstained with the use of nuclear fast red.

For neurogenic differentiation, DPSCs were cultured in Mesenchymal Stem Cell Neurogenic Differentiation Medium (C28015, PromoCell, Germany) for 10 days. Their neurogenic phenotypes were validated by the expression of mature neuron markers Tuj1 (1:200, Alexa Fluor 647 anti-Tubulin beta 3, 657406, BioLegend) and neurofilament-M (NEFM; 1:250, MA5-32613, ThermoFisher Scientific) visualized by confocal microscopy.

- 1. **Flow cytometry analysis**

The expression of MSC markers, pericyte and endothelial markers, as well as NOTCH 1-4, was evaluated by flow cytometry. The cells stained for intracellular markers were fixed and permeabilized in ice-cold methanol for 2 minutes at -20°C. The cells stained for NOTCH1-4 were fixed in 2% PFA for 15 minutes followed by permeabilization (Permeabilization buffer, 00-8333-56, Invitrogen, USA) in accordance with the manufacturer’s protocol. The samples containing 1 million cells/100 μl were then stained with fluorochrome-conjugated primary antibodies in a staining buffer (BUF073, Bio-Rad, USA) containing 0.1% BSA for 45 minutes on ice, followed by three washes in the buffer. Antibodies and isotype controls used for the analysis are listed in Appendix table 1. The samples were analyzed by Accuri C6 (BD BioSciences, USA) and visualized by FlowJo v10 (Becton, Dickinson & Company, USA).

- 1. **CD surface marker profiling and bioinformatics**

The CD marker expression profile of naiveDPSCs and endoDPSCs from 4 donors were evaluated by the antibody-based SocioCD microarray. Proteins were extracted with scioExtract buﬀer (Sciomics) using the manufacturer’s extraction SOPs. The bulk protein concentration was determined by BCA assay (Appendix Table 3). A reference sample was established by pooling an identical volume of each sample. The samples were labelled at an adjusted protein concentration for two hours with scioDye 2 (Sciomics). The reference sample was labelled with scioDye 1 (Sciomics). After two hours the reaction was stopped and the buﬀer exchanged to PBS. All labelled protein samples were stored at -20° C until use.

The samples were analyzed in a dual-color approach using a reference-based design on eight scioCD antibody microarrays (Sciomics) targeting diﬀerent CD surface markers. Each antibody is represented on the array in four replicates. The arrays were blocked with scioBlock (Sciomics) on a Hybstation 4800 (Tecan, Austria) and afterwards the samples were incubated competitively with the reference sample using a dual-color approach. After incubation for three hours, the slides were thoroughly washed with 1x PBSTT, rinsed with 0.1x PBS as well as with water and subsequently dried with nitrogen. Slide scanning was conducted using a Powerscanner (Tecan, Austria) with constant instrument laser power and PMT settings. Spot segmentation was performed with GenePix Pro 6.0 (Molecular Devices, Union City, CA, USA). Acquired raw data were analyzed using the linear models for microarray data (LIMMA) package of R-Bioconductor after uploading the median signal intensity. For normalization, a specialized invariant Lowess method was applied [1].

For data analysis, a multi-factorial linear model was fitted via least squares regression with LIMMA, resulting in a two-sided t-test or F-test based on moderated statistics. Next to the treatment factor, donor matching was accounted for using the information as an additional factor in the linear model. All presented p values were adjusted for multiple testing by controlling the false discovery rate according to Benjamini and Hochberg [2].

- 1. **Chorioallantoic membrane assay and vessel quantification**

Chorioallantoic membrane (CAM) assay was conducted to evaluate the pro-angiogenic and vasculogenic capacities of naiveDPSCs and endoDPSCs. Freshly fertilized *Gallus gallus domesticus* eggs were sourced from a local farm and incubated at 37.5 °C in a humidified environment for three days using a rotating egg incubator. The egg contents, including the developing embryos, were then transferred on embryonic day 3 into sterile weighing boats containing 2 ml of PBS and incubated for an additional four days at 37.5 °C. On embryonic day 7, Teflon rings in the dimension of 6.08 mm (ID) × 1.78 mm (H) (TBS2501-010, Tribi Science, USA) were placed on CAM so that no major vessels were disrupted. 500,000 cells of endoDPSCs or naiveDPSCs each were implanted in 50 μl of GFR-Matrigel onto the CAM within the Teflon ring and incubated for an additional 5 days before vessel quantification and dissection for histological assays.

On embryonic day 12, the CAM was dissected and fixed overnight in 4% PFA. for blood vessel quantification, the microscopic images were obtained using a stereomicroscope (M205C/MC170HD, Leica, Germany) and quantified using a deep learning-based image analysis software, IKOSA CAM Assay Application (KML Vision GmbH, Austria). For histological analysis, immunofluorescence followed by confocal microscopy and Paraffin-embedded tissue sectioning followed by hematoxylin-Eosin staining were conducted as previously described [3].

**Supplementary Figure**

**
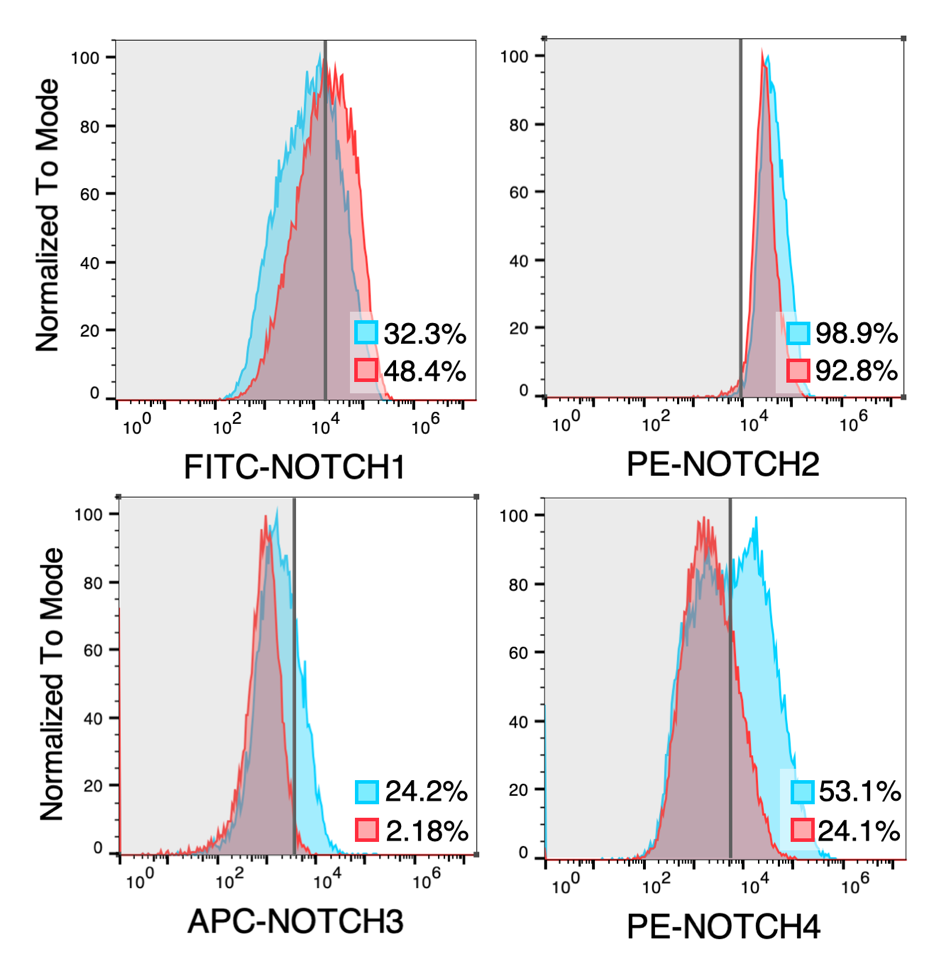
Fig S1. NOTCH expression in naiveDPSCs and endoDPSCs.** Flow cytometry analysis of NOTCH1-4. After endothelial induction, DPSCs notably altered their NOTCH expression, with NOTCH1-expressing population increased and NOTCH2-4 decreased.


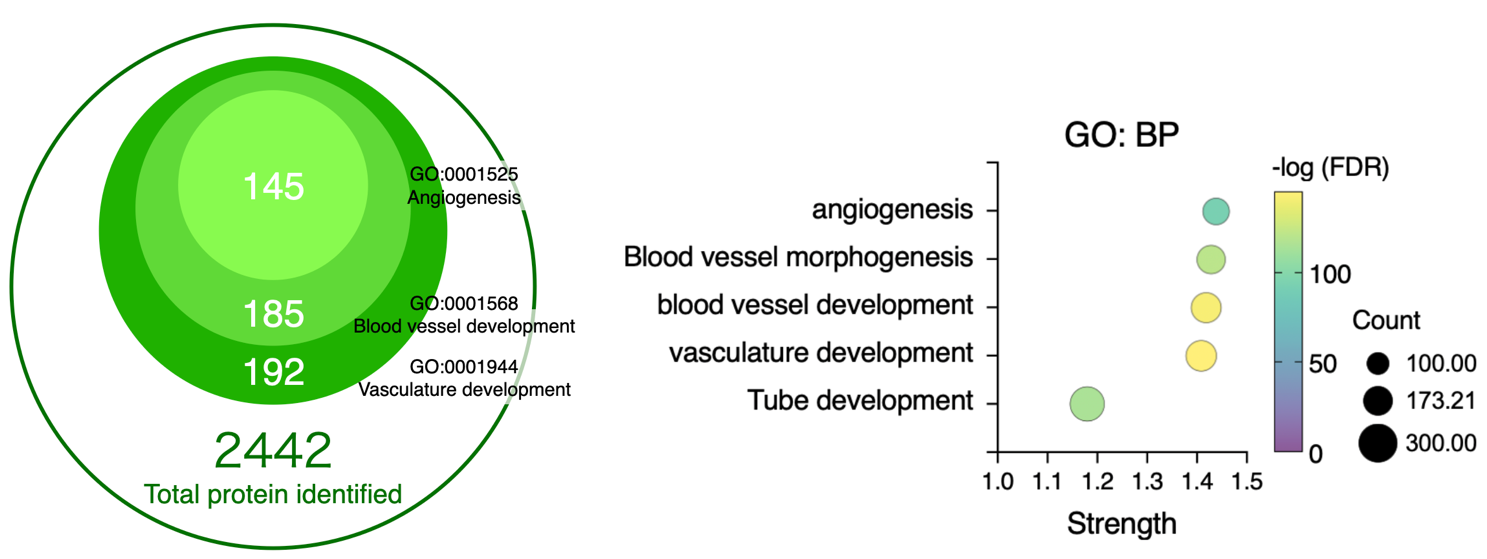


**Fig. S2 Mass-spectroscopy-based proteomics analysis of secretome from DPSCs**. Among 2442 proteins identified, 192 proteins were predicted to be involved in vasculature development, 185 were in blood vessel development, 145 were in angiogenesis (left). Gene Ontology (GO) analysis of identified proteins (right). Methodology of the proteomics is described previously (Yamada et al. 2024).

1. **Appendix Tables**

**Appendix Table 1. A list of antibodies and isotype controls used for flow cytometry analysis**

| **Antibodies (Conjugation)** | **Isotype** | **Dilution** | **Cat. No** | **Supplier** |
| --- | --- | --- | --- | --- |
| CD34 (PE) | Ms IgG1 | 1:100 | CD34-581-04 | Invitrogen |
| CD45 (PE) | Ms IgG1,κ | 1:100 | 368509 | BioLegend |
| HLA-DR (PE) | Ms IgG2a,κ | 1:100 | 555812 | BD Pharmingen |
| CD73 (FITC) | Ms IgG1,κ | 1:100 | 561254 | BD Pharmingen |
| CD90 (PerCP-Cy5.5) | Ms IgG1,κ | 1:250 | 561557 | BD Pharmingen |
| CD105 (PE) | Ms IgG1,κ | 1:250 | 560839 | BD Pharmingen |
| Axin2 | Rb Polyclonal | 1:250 | PA5-76674 | Invitrogen |
| Sox2 (AF488) | Ms IgG2a | 1:200 | 560301 | BD Pharmingen |
| Stem cell factor (FITC) | Rb Polyclonal | 1:250 | bs-0545R-FITC | Bioss |
| c-kit (APC) | Rt IgG2b | 1:250 | 17-1171-82 | Invitrogen |
| αSMA (AF488) | Ms IgG2a,κ | 1:250 | 53-9760-82 | Invitrogen |
| PDGFRβ (BF647) | Rb Polyclonal | 1:250 | bs-3322R-BF647 | Bioss |
| NG2 (AF488) | Ms IgG2a,κ | 1:200 | 53-6504-82 | Invitrogen |
| CD146 (eFluor660) | Ms IgG1,κ | 1:200 | 50-1469-42 | Invitrogen |
| Nestin (CL488) | Rb Polyclonal | 1:200 | CL488-19483 | ProteinTeck |
| CD31 (APC) | Ms IgG1,κ | 1:100 | 17-0319-42 | Invitrogen |
| CD144 (PE) | Ms IgG1,κ | 1:100 | 12-1449-82 | Invitrogen |
| wVF (PE) | Ms IgG1,κ | 1:100 | NBP2-34510PE | Novus |
| VEGFR1 (PE) | Ms IgG1 | 1:10 | FAB321P | Novus |
| VEGFR2 (AF488) | Ms IgG1,κ | 1:20 | 359913 | BioLegend |
| NOTCH1 (FITC) | Ms IgG1,κ | 1:10 | MA5-16862 | Invitrogen |
| NOTCH2 (PE) | Rt IgG1,κ | 1:20 | 12-5786-82 | Invitrogen |
| NOTCH 3 (APC) | Ms IgG1,κ | 1:20 | 17-5787-42 | Invitrogen |
| NOTCH 4 (PE) | Ms IgG1,κ | 1:20 | 563269 | BD Pharmingen |
| Isotype control (PE) | Ms IgG1,κ | - | 400111 | BioLegend |
| Isotype control (FITC) | Ms IgG1,κ | - | 554679 | BD Pharmingen |
| Isotype control (PerCP-Cy5.5) | Ms IgG1,κ | - | 552834 | BD Pharmingen |
| Isotype control (FITC) | Ms IgG2a,κ | - | 53-4724-80 | Invitrogen |
| Isotype control (APC) | Ms IgG1,κ | - | 17-4714-42 | Invitrogen |

**Appendix Table 2. A list of primers selected for customized TaqMan RT-qPCR array**

| **Assay ID** | **Gene Symbol** | **Gene Name(s)** | **Amplicon length (bp)** |
| --- | --- | --- | --- |
| Hs99999901_s1 | 18s rRNA | - | 0 |
| Hs99999905_m1 | GAPDH | - | 0 |
| Hs99999909_m1 | HPRT | - | 0 |
| Hs99999908_m1 | GUSB | - | 0 |
| Hs01060665_g1 | ACTB | actin beta | 63 |
| Hs00164932_m1 | ICAM1 | intercellular adhesion molecule 1 | 87 |
| Hs01065279_m1 | PECAM1 | platelet and endothelial cell adhesion molecule 1 | 78 |
| Hs01003372_m1 | VCAM1 | vascular cell adhesion molecule 1 | 62 |
| Hs01109446_m1 | VWF | von Willebrand factor | 56 |
| Hs01651836_m1 | FAM174B | family with sequence similarity 174 member B | 62 |
| Hs00373501_m1 | ADAMTS18 | ADAM metallopeptidase with thrombospondin type 1 motif 18 | 81 |
| Hs00232660_m1 | LHX6 | LIM homeobox 6 | 78 |
| Hs00187290_m1 | NRP2 | neuropilin 2 | 81 |
| Hs01119113_m1 | EPHB4 | EPH receptor B4 | 73 |
| Hs00826128_m1 | NRP1 | neuropilin 1 | 90 |
| Hs01012057_m1 | HEY2 | hes related family bHLH transcription factor with YRPW motif 2 | 90 |
| Hs00216777_m1 | ANTXR1 | anthrax toxin receptor 1 | 67 |
| Hs01070009_m1 | RASGRF2 | Ras protein specific guanine nucleotide releasing factor 2 | 59 |
| Hs01062014_m1 | NOTCH1 | notch 1 | 80 |
| Hs01052961_m1 | FLT1 | fms related tyrosine kinase 1 | 72 |
| Hs00911700_m1 | KDR | kinase insert domain receptor | 83 |
| Hs01047677_m1 | FLT4 | fms related tyrosine kinase 4 | 111 |
| Hs01009259_m1 | PROM1 | prominin 1 | 66 |
| Hs02576480_m1 | CD34 | CD34 molecule | 63 |
| Hs00174961_m1 | EDN1 | endothelin 1 | 62 |
| Hs01012714_m1 | EDN2 | endothelin 2 | 127 |
| Hs03988672_m1 | EDNRA | endothelin receptor type A | 96 |
| Hs00950401_m1 | SELE | selectin E | 104 |
| Hs00900055_m1 | VEGFA | vascular endothelial growth factor A | 59 |
| Hs00174029_m1 | KIT | KIT proto-oncogene receptor tyrosine kinase | 64 |
| Hs00936295_m1 | BSG | basigin (Ok blood group) | 97 |
| Hs01075529_m1 | NOS2 | nitric oxide synthase 2 | 67 |
| Hs01574659_m1 | NOS3 | nitric oxide synthase 3 | 107 |
| Hs00173590_m1 | NPPB | natriuretic peptide B | 82 |
| Hs00181445_m1 | NPR1 | natriuretic peptide receptor 1 | 75 |
| Hs00901463_m1 | CDH5 | cadherin 5 | 63 |
| Hs00157317_m1 | TYMP | thymidine phosphorylase | 95 |
| Hs00540548_s1 | CXCR5 | C-X-C motif chemokine receptor 5 | 73 |
| Hs00174575_m1 | CCL5 | C-C motif chemokine ligand 5 | 63 |
| Hs00234140_m1 | CCL2 | C-C motif chemokine ligand 2 | 101 |
| Hs00171086_m1 | CX3CL1 | C-X3-C motif chemokine ligand 1 | 72 |
| Hs00174151_m1 | SELL | selectin L | 62 |
| Hs00380945_m1 | SELPLG | selectin P ligand | 68 |
| Hs00919949_m1 | PTGIS | prostaglandin I2 (prostacyclin) synthase | 56 |
| Hs01070189_m1 | BST1 | bone marrow stromal cell antigen 1 | 83 |
| Hs00170162_m1 | OCLN | occludin | 68 |
| Hs01009821_m1 | ADAMTS13 | ADAM metallopeptidase with thrombospondin type 1 motif 13 | 86 |
| Hs00174179_m1 | ACE | angiotensin I converting enzyme | 74 |

**Appendix Table 3. Protein concentration in the cell lysate determined by BCA assay for the SocioCD microarray**

| Donor | Condition | Protein concentration (mg/ml) |
| --- | --- | --- |
| 1 | naiveDPSC | 1.84 |
| 2 | naiveDPSC | 1.76 |
| 3 | naiveDPSC | 2.01 |
| 4 | naiveDPSC | 1.97 |
| 1 | endoDPSC | 1.86 |
| 2 | endoDPSC | 2.01 |
| 3 | endoDPSC | 1.65 |
| 4 | endoDPSC | 2.02 |

**Appendix Table 4. The antibody panel socioCD microarray and acquired data**

|  |  |  |  |  | **endoDPSCs vs naiveDPSCs** | | |
| --- | --- | --- | --- | --- | --- | --- | --- |
| **Antibody ID** | **Name** | **CD Marker** | **HGNC** | **Ave.Exp.** | **log2FC** | **p-value** | **adj.p-val** |
| ab1380 | NEP | CD10 | MME | 7.99 | -0.072 | 2.5E-01 | 4.8E-01 |
| ab1381 | NEP | CD10 | MME | 9.33 | 0.036 | 3.7E-01 | 6.1E-01 |
| ab2254 | NEP | CD10 | MME | 9.63 | -0.585 | 4.2E-13 | 1.1E-11 |
| ab1573 | ITAE | CD103 | ITGAE | 8.05 | -0.147 | 3.4E-03 | 1.7E-02 |
| ab1483 | EGLN | CD105 | ENG | 10.20 | -0.527 | 3.6E-11 | 7.6E-10 |
| ab1484 | EGLN | CD105 | ENG | 10.47 | -0.661 | 2.7E-14 | 9.2E-13 |
| ab1746 | EGLN | CD105 | ENG | 11.14 | -0.231 | 1.1E-02 | 4.5E-02 |
| ab1792 | VCAM1 | CD106 | VCAM1 | 11.19 | -0.475 | 9.0E-03 | 3.8E-02 |
| ab1625 | LAMP1 | CD107a | LAMP1 | 9.59 | -0.020 | 7.8E-01 | 9.1E-01 |
| ab1574 | SEM7A | CD108 | SEMA7A | 7.35 | -0.001 | 9.7E-01 | 9.8E-01 |
| ab2506 | CSF3R | CD114 | CSF3R | 7.55 | -0.002 | 9.6E-01 | 9.8E-01 |
| ab1925 | CSF1R | CD115 | CSF1R | 10.10 | -0.090 | 5.6E-02 | 1.8E-01 |
| ab1384 | ITAL | CD11a | ITGAL | 9.27 | 0.122 | 6.8E-01 | 8.6E-01 |
| ab1385 | ITAL | CD11a | ITGAL | 8.71 | 0.363 | 1.0E-01 | 2.7E-01 |
| ab1387 | ITAM | CD11b | ITGAM | 6.69 | 0.061 | 6.9E-01 | 8.6E-01 |
| ab1388 | ITAM | CD11b | ITGAM | 8.37 | 0.039 | 4.3E-01 | 6.6E-01 |
| ab1389 | ITAX | CD11c | ITGAX | 7.97 | 0.007 | 8.9E-01 | 9.6E-01 |
| ab2332 | TNR1A | CD120a | TNFRSF1A | 13.11 | 0.222 | 2.0E-03 | 1.1E-02 |
| ab1610 | TNR1A | CD120a | TNFRSF1A | 7.57 | 0.176 | 1.5E-02 | 5.9E-02 |
| ab2455 | TNR1B | CD120b | TNFRSF1B | 7.43 | 0.163 | 5.3E-02 | 1.7E-01 |
| ab2480 | IL1R2 | CD121 | IL1R2 | 7.05 | -0.070 | 2.1E-01 | 4.3E-01 |
| ab2100 | IL1R1 | CD121A | IL1R1 | 8.28 | -0.019 | 8.6E-01 | 9.4E-01 |
| ab1575 | IL2RB | CD122 | IL2RB | 7.27 | 0.040 | 4.8E-01 | 7.1E-01 |
| ab2041 | IL3RA | CD123 | IL3RA | 7.59 | 0.012 | 8.9E-01 | 9.6E-01 |
| ab2433 | IL6RA | CD126 | IL6R | 7.35 | -0.021 | 7.6E-01 | 9.0E-01 |
| ab1390 | AMPN | CD13 | ANPEP | 9.55 | 0.026 | 6.1E-01 | 8.1E-01 |
| ab2383 | AMPN | CD13 | ANPEP | 9.81 | 0.692 | 1.3E-15 | 5.4E-14 |
| ab2315 | IL6RB | CD130 | IL6ST | 10.11 | 0.237 | 5.2E-04 | 3.4E-03 |
| ab1551 | IL3RB | CD131 | CSF2RB | 9.11 | -0.045 | 2.4E-01 | 4.6E-01 |
| ab3678 | TNR4 | CD134 | TNFRSF4 | 11.49 | 0.144 | 2.9E-03 | 1.5E-02 |
| ab1980 | FLT3 | CD135 | FLT3 | 8.19 | -0.044 | 4.1E-01 | 6.5E-01 |
| ab2274 | TNR9 | CD137 | TNFRSF9 | 8.99 | 0.003 | 9.5E-01 | 9.8E-01 |
| ab1028 | TNR9 | CD137 | TNFRSF9 | 10.11 | 0.047 | 2.9E-01 | 5.4E-01 |
| ab1576 | SDC1 | CD138 | SDC1 | 8.26 | -0.191 | 4.2E-04 | 2.8E-03 |
| ab2308 | SDC1 | CD138 | SDC1 | 8.48 | -0.233 | 6.0E-05 | 4.6E-04 |
| ab1393 | CD14 | CD14 | CD14 | 8.21 | 0.017 | 6.6E-01 | 8.5E-01 |
| ab2267 | CD14 | CD14 | CD14 | 10.14 | 0.688 | 3.5E-09 | 5.0E-08 |
| ab2202 | TF | CD142 | F3 | 10.93 | 0.907 | 1.8E-07 | 1.9E-06 |
| ab2818 | CADH5 | CD144 | CDH5 | 14.75 | -0.116 | 8.6E-02 | 2.4E-01 |
| ab1486 | BASI | CD147 | BSG | 10.00 | -0.116 | 7.9E-03 | 3.4E-02 |
| ab1487 | BASI | CD147 | BSG | 12.62 | -0.361 | 7.6E-08 | 8.7E-07 |
| ab1637 | BASI | CD147 | BSG | 11.18 | 0.139 | 8.0E-04 | 4.9E-03 |
| ab1910 | BASI | CD147 | BSG | 12.09 | -0.312 | 1.2E-08 | 1.5E-07 |
| ab1394 | CD15 | CD15 |  | 6.99 | 0.032 | 5.2E-01 | 7.4E-01 |
| ab1395 | CD15 | CD15 |  | 7.76 | -0.054 | 3.2E-01 | 5.7E-01 |
| ab1033 | SLAF1 | CD150 | SLAMF1 | 8.18 | -0.095 | 4.1E-02 | 1.4E-01 |
| ab2132 | SLAF1 | CD150 | SLAMF1 | 12.65 | 0.467 | 3.2E-09 | 4.9E-08 |
| ab1117 | CTLA4 | CD152 | CTLA4 | 11.44 | -0.094 | 2.3E-01 | 4.6E-01 |
| ab1171 | TNFL8 | CD153 | TNFSF8 | 7.90 | 0.058 | 2.0E-01 | 4.1E-01 |
| ab1936 | ADAM8 | CD156 | ADAM8 | 7.16 | 0.027 | 6.8E-01 | 8.6E-01 |
| ab2342 | ADA17 | CD156B | ADAM17 | 12.81 | 0.872 | 2.5E-11 | 5.4E-10 |
| ab1134 | KI2L2 | CD158b | KIR2DL2 | 7.92 | 0.203 | 5.9E-03 | 2.7E-02 |
| ab1397 | FCG3A | CD16 | FCGR3A | 6.47 | 0.029 | 7.3E-01 | 8.8E-01 |
| ab1532 | FCG3A | CD16 | FCGR3A | 8.39 | -0.051 | 6.0E-01 | 8.0E-01 |
| ab1488 | SELPL | CD162 | SELPLG | 9.67 | 0.086 | 7.8E-03 | 3.4E-02 |
| ab1944 | C163A | CD163 | CD163 | 14.15 | 0.849 | 7.0E-15 | 2.7E-13 |
| ab2246 | CD166 | CD166 | ALCAM | 13.20 | -0.426 | 1.6E-06 | 1.5E-05 |
| ab1400 | CD17 | CD17 |  | 7.69 | 0.077 | 8.4E-02 | 2.3E-01 |
| ab1489 | CD177 | CD177 | CD177 | 8.37 | -0.009 | 8.3E-01 | 9.4E-01 |
| ab1744 | KIT | CD177 | KIT | 8.10 | -0.027 | 5.4E-01 | 7.6E-01 |
| ab1983 | TNFL6 | CD178 | FASLG | 8.19 | 0.098 | 5.9E-02 | 1.8E-01 |
| ab1402 | ITB2 | CD18 | ITGB2 | 8.56 | 0.132 | 6.3E-02 | 1.9E-01 |
| ab1404 | ITB2 | CD18 | ITGB2 | 8.15 | -0.041 | 4.2E-01 | 6.5E-01 |
| ab3667 | CXCR5 | CD185 | CXCR5 | 9.99 | 0.112 | 2.4E-03 | 1.3E-02 |
| ab1405 | CD19 | CD19 | CD19 | 7.90 | -0.076 | 1.7E-01 | 3.7E-01 |
| ab1406 | CD19 | CD19 | CD19 | 8.78 | -0.038 | 4.3E-01 | 6.6E-01 |
| ab0987 | CCR7 | CD197 | CCR7 | 8.85 | 0.071 | 7.7E-01 | 9.0E-01 |
| ab1353 | CD1A | CD1a | CD1A | 9.65 | -0.011 | 9.5E-01 | 9.8E-01 |
| ab1355 | CD2 | CD2 | CD2 | 8.13 | 0.205 | 3.1E-04 | 2.1E-03 |
| ab1356 | CD2 | CD2 | CD2 | 12.55 | 0.327 | 2.7E-03 | 1.4E-02 |
| ab1408 | CD20 | CD20 | MS4A1 | 9.17 | -0.019 | 6.2E-01 | 8.1E-01 |
| ab1594 | CD20 | CD20 | MS4A1 | 9.50 | 0.190 | 4.2E-03 | 2.0E-02 |
| ab1754 | OX2G | CD200 | CD200 | 7.76 | 0.121 | 5.9E-02 | 1.8E-01 |
| ab2135 | EPCR | CD201 | PROCR | 8.49 | -0.145 | 4.0E-03 | 1.9E-02 |
| ab1882 | TIE2 | CD202b | TEK | 7.55 | -0.010 | 9.5E-01 | 9.8E-01 |
| ab3674 | LY75 | CD205 | LY75 | 7.67 | 0.009 | 8.7E-01 | 9.5E-01 |
| ab0988 | CR2 | CD21 | CR2 | 8.94 | 0.257 | 3.5E-03 | 1.7E-02 |
| ab1410 | CR2 | CD21 | CR2 | 9.67 | 0.035 | 4.2E-01 | 6.6E-01 |
| ab1533 | CR2 | CD21 | CR2 | 8.60 | 0.021 | 7.2E-01 | 8.8E-01 |
| ab1970 | I13R1 | CD213a1 | IL13RA1 | 9.58 | 0.099 | 1.2E-02 | 4.6E-02 |
| ab1947 | I13R2 | CD213a2 | IL13RA2 | 13.32 | -0.340 | 2.4E-07 | 2.5E-06 |
| ab2407 | I17RA | CD217 | IL17RA | 11.38 | 0.136 | 4.4E-01 | 6.7E-01 |
| ab1412 | CD22 | CD22 | CD22 | 7.69 | 0.061 | 7.4E-01 | 8.9E-01 |
| ab1413 | CD22 | CD22 | CD22 | 8.00 | -0.032 | 5.7E-01 | 7.8E-01 |
| ab1995 | IGF1R | CD221 | IGF1R | 9.38 | -0.087 | 7.3E-02 | 2.1E-01 |
| ab1490 | MPRI | CD222 | IGF2R | 9.73 | -0.077 | 1.6E-01 | 3.6E-01 |
| ab1915 | MPRI | CD222 | IGF2R | 10.96 | 0.229 | 8.8E-03 | 3.7E-02 |
| ab2794 | LAG3 | CD223 | LAG3 | 8.65 | 0.032 | 6.7E-01 | 8.6E-01 |
| ab1087 | MUC1 | CD227 | MUC1 | 11.28 | -0.500 | 4.7E-09 | 6.4E-08 |
| ab1414 | FCER2 | CD23 | FCER2 | 8.19 | 0.014 | 8.2E-01 | 9.3E-01 |
| ab1900 | FCER2 | CD23 | FCER2 | 7.81 | 0.040 | 3.6E-01 | 6.1E-01 |
| ab1345 | PRIO | CD230 | PRNP | 14.23 | -0.351 | 6.9E-11 | 1.3E-09 |
| ab1491 | GLPA | CD235a | GYPA | 8.13 | -0.161 | 5.0E-03 | 2.3E-02 |
| ab1636 | CD235a | CD235a | GYPA | 8.65 | 0.024 | 7.5E-01 | 9.0E-01 |
| ab1492 | GLPB | CD235b | GYPB | 10.00 | 0.079 | 9.4E-02 | 2.5E-01 |
| ab2290 | BCAM | CD239 | BCAM | 11.10 | 1.094 | 1.9E-20 | 1.6E-18 |
| ab1415 | CD24 | CD24 | CD24 | 9.10 | 0.067 | 9.9E-02 | 2.6E-01 |
| ab2157 | CD244 | CD244 | CD244 | 8.24 | -0.127 | 3.4E-02 | 1.2E-01 |
| ab1417 | IL2RA | CD25 | IL2RA | 8.46 | 0.050 | 2.3E-01 | 4.6E-01 |
| ab1418 | IL2RA | CD25 | IL2RA | 10.41 | -0.603 | 4.2E-06 | 3.8E-05 |
| ab1831 | IL2RA | CD25 | IL2RA | 13.34 | 0.098 | 2.2E-01 | 4.5E-01 |
| ab3560 | TNFL4 | CD252 |  | 12.36 | -0.079 | 1.3E-01 | 3.1E-01 |
| ab2073 | TNF10 | CD253 | TNFSF10 | 7.53 | -0.026 | 5.9E-01 | 7.9E-01 |
| ab1663 | TNF10 | CD253 | TNFSF10 | 7.54 | -0.042 | 3.5E-01 | 6.0E-01 |
| ab2674 | TNF11 | CD254 | TNFSF11 | 15.17 | 0.011 | 9.1E-01 | 9.7E-01 |
| ab1086 | TNF13 | CD256 | TNFSF13 | 8.24 | 0.084 | 5.1E-02 | 1.7E-01 |
| ab2788 | TNF13 | CD256 | TNFSF13 | 7.66 | -0.004 | 9.3E-01 | 9.7E-01 |
| ab1711 | TN13B | CD257 | TNFSF13B | 10.94 | 0.069 | 1.2E-01 | 2.9E-01 |
| ab2736 | TN13B | CD257 | TNFSF13B | 7.97 | -0.011 | 7.5E-01 | 9.0E-01 |
| ab2301 | TNF14 | CD258 | TNFSF14 | 8.08 | -0.021 | 5.9E-01 | 7.9E-01 |
| ab1558 | DPP4 | CD26 | DPP4 | 8.73 | 0.003 | 9.5E-01 | 9.8E-01 |
| ab1778 | DPP4 | CD26 | DPP4 | 9.05 | -0.224 | 2.5E-02 | 9.0E-02 |
| ab1794 | TR10D | CD264 | TNFRSF10D | 9.42 | 0.085 | 8.0E-02 | 2.3E-01 |
| ab1857 | TNR11 | CD265 | TNFRSF11A | 15.65 | -0.140 | 2.9E-03 | 1.5E-02 |
| ab2250 | TR13B | CD267 | TNFRSF13B | 9.80 | 0.050 | 1.6E-01 | 3.6E-01 |
| ab3679 | TR13C | CD268 | TNFRSF13C | 8.26 | -0.108 | 1.3E-01 | 3.0E-01 |
| ab2227 | TNR17 | CD269 | TNFRSF17 | 8.13 | -0.108 | 1.5E-02 | 5.9E-02 |
| ab3666 | CD27 | CD27 | CD27 | 8.84 | 0.267 | 1.7E-03 | 9.4E-03 |
| ab1765 | TNR14 | CD270 | TNFRSF14 | 8.17 | -0.027 | 6.9E-01 | 8.7E-01 |
| ab2445 | TNR16 | CD271 | NGFR | 12.54 | -0.061 | 4.4E-01 | 6.7E-01 |
| ab3663 | BTLA | CD272 | BTLA | 11.17 | -0.148 | 9.6E-02 | 2.6E-01 |
| ab2734 | PD1L2 | CD273 | PDCD1LG2 | 7.80 | 0.021 | 7.8E-01 | 9.1E-01 |
| ab1823 | PD1L1 | CD274 | CD274 | 10.84 | 0.098 | 1.2E-01 | 2.9E-01 |
| ab2437 | CD276 | CD276 | CD276 | 10.26 | 0.009 | 7.8E-01 | 9.1E-01 |
| ab2742 | ICOS | CD278 | ICOS | 12.59 | -0.083 | 3.7E-01 | 6.1E-01 |
| ab1352 | PDCD1 | CD279 | PDCD1 | 7.96 | 0.001 | 9.9E-01 | 9.9E-01 |
| ab1420 | CD28 | CD28 | CD28 | 8.06 | 0.042 | 3.2E-01 | 5.6E-01 |
| ab1559 | CD28 | CD28 | CD28 | 8.33 | -0.043 | 3.9E-01 | 6.4E-01 |
| ab1868 | TLR2 | CD282 | TLR2 | 8.67 | -0.081 | 6.5E-02 | 2.0E-01 |
| ab2700 | TLR3 | CD283 | TLR3 | 13.10 | -0.060 | 3.1E-01 | 5.5E-01 |
| ab1534 | ITB1 | CD29 | ITGB1 | 12.28 | 0.071 | 4.1E-01 | 6.5E-01 |
| ab2812 | LEPR | CD295 | LEPR | 7.90 | -0.002 | 9.8E-01 | 9.9E-01 |
| ab1423 | TNR8 | CD30 | TNFRSF8 | 9.27 | 0.095 | 1.4E-01 | 3.3E-01 |
| ab2743 | TNR8 | CD30 | TNFRSF8 | 12.09 | 0.398 | 1.1E-04 | 8.1E-04 |
| ab2075 | VGFR2 | CD309 | KDR | 6.89 | 0.023 | 7.8E-01 | 9.1E-01 |
| ab1424 | PECA1 | CD31 | PECAM1 | 8.42 | 0.046 | 2.3E-01 | 4.6E-01 |
| ab1535 | PECA1 | CD31 | PECAM1 | 8.43 | 0.148 | 6.2E-02 | 1.9E-01 |
| ab2473 | PECA1 | CD31 | PECAM1 | 8.26 | 0.056 | 3.3E-01 | 5.7E-01 |
| ab2813 | CDCP1 | CD318 | CDCP1 | 10.15 | 0.759 | 9.8E-08 | 1.1E-06 |
| ab1561 | FCG2A | CD32 | FCGR2A | 8.52 | 0.306 | 2.0E-05 | 1.7E-04 |
| ab1803 | FCG2B | CD32 | FCGR2B | 7.41 | 0.021 | 6.6E-01 | 8.5E-01 |
| ab2412 | CADH1 | CD324 | CDH1 | 8.85 | 0.147 | 4.7E-02 | 1.6E-01 |
| ab2760 | CADH2 | CD325 | CDH2 | 13.62 | 0.526 | 4.4E-09 | 6.1E-08 |
| ab1987 | EPCAM | CD326 | EPCAM | 8.25 | 0.095 | 1.4E-01 | 3.2E-01 |
| ab2005 | SIGL9 | CD329 | SIGLEC9 | 6.99 | 0.057 | 2.6E-01 | 4.9E-01 |
| ab1425 | CD33 | CD33 | CD33 | 7.71 | -0.025 | 5.4E-01 | 7.6E-01 |
| ab1562 | CD33 | CD33 | CD33 | 8.03 | -0.121 | 7.6E-02 | 2.2E-01 |
| ab2718 | JAG1 | CD339 | JAG1 | 7.61 | 0.049 | 4.7E-01 | 7.0E-01 |
| ab1426 | CD34 | CD34 | CD34 | 8.25 | -0.047 | 2.2E-01 | 4.4E-01 |
| ab2784 | ERBB2 | CD340 | ERBB2 | 9.86 | 0.210 | 6.4E-04 | 4.1E-03 |
| ab3670 | ERBB2 | CD340 | ERBB2 | 8.22 | 0.078 | 1.5E-01 | 3.4E-01 |
| ab1536 | CR1 | CD35 | CR1 | 7.44 | -0.015 | 7.0E-01 | 8.7E-01 |
| ab2129 | SLAF8 | CD353 | SLAMF8 | 14.25 | 0.053 | 1.9E-01 | 4.1E-01 |
| ab2125 | TREM1 | CD354 | TREM1 | 8.17 | -0.108 | 7.5E-02 | 2.2E-01 |
| ab1892 | CRTAM | CD355 | CRTAM | 7.99 | -0.109 | 8.2E-02 | 2.3E-01 |
| ab2113 | TNR18 | CD357 | TNFRSF18 | 9.34 | 0.038 | 7.8E-01 | 9.1E-01 |
| ab2145 | TNR21 | CD358 | TNFRSF21 | 8.14 | -0.074 | 9.3E-02 | 2.5E-01 |
| ab1427 | CD36 | CD36 | CD36 | 7.44 | -0.019 | 7.1E-01 | 8.7E-01 |
| ab2783 | CD36 | CD36 | CD36 | 14.68 | -0.429 | 1.3E-09 | 2.1E-08 |
| ab2265 | HAVR1 | CD365 | HAVCR1 | 7.93 | -0.110 | 1.9E-02 | 7.2E-02 |
| ab2781 | HAVR1 | CD365 | HAVCR1 | 6.70 | 0.007 | 9.1E-01 | 9.7E-01 |
| ab2067 | HAVR2 | CD366 | HAVCR2 | 8.86 | -0.099 | 8.2E-02 | 2.3E-01 |
| ab2146 | SCRB2 | CD36L2 | SCARB2 | 8.62 | -0.094 | 6.9E-02 | 2.0E-01 |
| ab1428 | CD37 | CD37 | CD37 | 7.69 | -0.025 | 7.0E-01 | 8.7E-01 |
| ab1429 | CD38 | CD38 | CD38 | 8.16 | -0.118 | 4.2E-01 | 6.5E-01 |
| ab1537 | CD38 | CD38 | CD38 | 8.35 | 0.069 | 3.6E-01 | 6.1E-01 |
| ab1111 | ENTP1 | CD39 | ENTPD1 | 10.99 | -0.135 | 2.3E-02 | 8.6E-02 |
| ab1358 | CD3E | CD3e | CD3E | 7.82 | 0.028 | 5.0E-01 | 7.3E-01 |
| ab1359 | CD3E | CD3e | CD3E | 7.93 | -0.012 | 7.4E-01 | 8.9E-01 |
| ab1524 | CD3deg | CD3δ | CD3E | 10.50 | -0.089 | 5.5E-01 | 7.7E-01 |
| ab1362 | CD4 | CD4 | CD4 | 7.35 | 0.199 | 9.5E-03 | 3.9E-02 |
| ab1525 | CD4 | CD4 | CD4 | 7.98 | 0.056 | 1.7E-01 | 3.6E-01 |
| ab1626 | CD4 | CD4 | CD4 | 9.03 | -0.078 | 1.3E-01 | 3.2E-01 |
| ab1430 | TNR5 | CD40 | CD40 | 9.00 | 0.065 | 7.2E-01 | 8.8E-01 |
| ab1752 | TNR5 | CD40 | CD40 | 9.02 | 0.009 | 8.6E-01 | 9.4E-01 |
| ab2122 | CD40L | CD40LG | CD40LG | 8.18 | -0.100 | 1.0E-01 | 2.7E-01 |
| ab1432 | ITA2B | CD41a | ITGA2B | 8.36 | 0.082 | 1.0E-01 | 2.7E-01 |
| ab1538 | ITA2B | CD41a | ITGA2B | 11.96 | 0.839 | 9.9E-09 | 1.3E-07 |
| ab1539 | ITA2B | CD41a | ITGA2B | 7.74 | 0.006 | 9.4E-01 | 9.8E-01 |
| ab1563 | ITA2B | CD41a | ITGA2B | 7.89 | -0.041 | 3.9E-01 | 6.4E-01 |
| ab1433 | GP1BA | CD42b | GP1BA | 12.98 | -0.440 | 1.1E-04 | 8.0E-04 |
| ab1434 | LEUK | CD43 | SPN | 9.42 | -0.003 | 9.2E-01 | 9.7E-01 |
| ab1435 | LEUK | CD43 | SPN | 8.46 | 0.012 | 8.2E-01 | 9.3E-01 |
| ab0991 | LEUK | CD43 | SPN | 10.94 | 0.073 | 8.1E-02 | 2.3E-01 |
| ab1437 | CD44 | CD44 | CD44 | 10.79 | -1.019 | 5.5E-22 | 5.6E-20 |
| ab1540 | CD44 | CD44 | CD44 | 11.20 | -0.574 | 4.3E-17 | 2.4E-15 |
| ab2782 | CD44v2 | CD44 | CD44 | 13.79 | -1.003 | 3.3E-20 | 2.4E-18 |
| ab1439 | PTPRC | CD45 | PTPRC | 7.56 | 0.038 | 3.1E-01 | 5.5E-01 |
| ab1440 | PTPRC | CD45 | PTPRC | 8.61 | 0.031 | 5.0E-01 | 7.3E-01 |
| ab1442 | CD45RA | CD45RA | PTPRC | 7.95 | 0.024 | 6.2E-01 | 8.1E-01 |
| ab1443 | CD45RA | CD45RA | PTPRC | 9.28 | 0.015 | 8.6E-01 | 9.4E-01 |
| ab1444 | CD45RB | CD45RB | PTPRC | 8.66 | -0.022 | 6.3E-01 | 8.2E-01 |
| ab1445 | CD45RO | CD45RO | PTPRC | 8.44 | -0.028 | 6.2E-01 | 8.1E-01 |
| ab1446 | MCP | CD46 | CD46 | 9.17 | -0.032 | 5.1E-01 | 7.4E-01 |
| ab1448 | CD47 | CD47 | CD47 | 13.90 | -0.125 | 3.7E-02 | 1.3E-01 |
| ab1542 | CD47 | CD47 | CD47 | 8.67 | 0.022 | 5.5E-01 | 7.7E-01 |
| ab2015 | CD47 | CD47 | CD47 | 9.21 | -0.349 | 1.6E-01 | 3.6E-01 |
| ab1449 | CD48 | CD48 | CD48 | 7.49 | -0.041 | 4.5E-01 | 6.7E-01 |
| ab1543 | ITA4 | CD49d | ITGA4 | 7.10 | 0.055 | 4.0E-01 | 6.4E-01 |
| ab1566 | ITA6 | CD49f | ITGA6 | 10.87 | 0.384 | 1.0E-05 | 8.8E-05 |
| ab1366 | CD5 | CD5 | CD5 | 8.47 | 0.088 | 5.5E-03 | 2.5E-02 |
| ab1529 | CD5 | CD5 | CD5 | 9.83 | 0.005 | 9.2E-01 | 9.7E-01 |
| ab1450 | ICAM3 | CD50 | ICAM3 | 11.87 | -0.102 | 5.3E-01 | 7.5E-01 |
| ab2009 | ICAM3 | CD50 | ICAM3 | 6.83 | -0.049 | 5.6E-01 | 7.7E-01 |
| ab1565 | ITA5 | CD51 | ITGA5 | 12.54 | 1.023 | 1.4E-13 | 3.9E-12 |
| ab1567 | ITAV | CD51 | ITGAV | 9.95 | -0.074 | 5.5E-02 | 1.8E-01 |
| ab1451 | CD52 | CD52 | CD52 | 8.31 | 0.016 | 9.6E-01 | 9.8E-01 |
| ab1453 | CD53 | CD53 | CD53 | 15.48 | 0.014 | 8.6E-01 | 9.4E-01 |
| ab1544 | CD53 | CD53 | CD53 | 13.19 | -0.026 | 7.1E-01 | 8.7E-01 |
| ab1454 | ICAM1 | CD54 | ICAM1 | 12.42 | -0.451 | 3.3E-09 | 4.9E-08 |
| ab1455 | ICAM1 | CD54 | ICAM1 | 12.36 | -0.429 | 1.0E-08 | 1.3E-07 |
| ab1864 | ICAM1 | CD54 | ICAM1 | 8.86 | -0.600 | 6.2E-12 | 1.5E-10 |
| ab1456 | DAF | CD55 | CD55 | 8.22 | -0.037 | 3.9E-01 | 6.4E-01 |
| ab1457 | DAF | CD55 | CD55 | 7.30 | -0.010 | 8.9E-01 | 9.6E-01 |
| ab1458 | NCAM1 | CD56 | NCAM1 | 7.54 | -0.019 | 6.7E-01 | 8.6E-01 |
| ab1459 | NCAM1 | CD56 | NCAM1 | 12.57 | -0.274 | 1.5E-07 | 1.6E-06 |
| ab2478 | NCAM1 | CD56 | NCAM1 | 8.75 | 0.105 | 2.4E-02 | 8.9E-02 |
| ab1460 | CD57 | CD57 | B3GAT1 | 7.36 | 0.062 | 2.5E-01 | 4.9E-01 |
| ab1568 | B3GA1 | CD57 | B3GAT1 | 8.01 | -0.120 | 7.8E-02 | 2.2E-01 |
| ab1461 | LFA3 | CD58 | CD58 | 8.68 | -0.015 | 8.0E-01 | 9.2E-01 |
| ab1462 | LFA3 | CD58 | CD58 | 8.81 | 0.068 | 1.2E-01 | 2.9E-01 |
| ab1463 | CD59 | CD59 | CD59 | 9.15 | 0.174 | 1.3E-03 | 7.2E-03 |
| ab1464 | CD59 | CD59 | CD59 | 11.10 | 0.246 | 3.0E-05 | 2.4E-04 |
| ab1367 | CD6 | CD6 | CD6 | 8.26 | -0.024 | 7.1E-01 | 8.7E-01 |
| ab1368 | CD6 | CD6 | CD6 | 7.64 | -0.002 | 9.7E-01 | 9.8E-01 |
| ab1569 | LYAM2 | CD62 | SELE | 14.40 | -0.037 | 6.6E-01 | 8.5E-01 |
| ab1943 | LYAM3 | CD62 | SELP | 14.98 | -0.156 | 1.4E-01 | 3.2E-01 |
| ab1999 | LYAM2 | CD62E | SELE | 13.39 | -0.344 | 3.4E-07 | 3.3E-06 |
| ab1466 | LYAM1 | CD62L | SELL | 7.59 | -0.261 | 5.5E-02 | 1.8E-01 |
| ab2189 | LYAM1 | CD62L | SELL | 7.32 | 0.049 | 5.7E-01 | 7.8E-01 |
| ab1570 | LYAM3 | CD62P | SELP | 8.21 | -0.179 | 1.1E-02 | 4.5E-02 |
| ab1468 | CD63 | CD63 | CD63 | 8.80 | 0.142 | 9.5E-03 | 3.9E-02 |
| ab1546 | CD63 | CD63 | CD63 | 9.45 | 0.347 | 3.6E-07 | 3.5E-06 |
| ab1628 | CEAM1,3 | CD66a | CEACAM1 | 8.86 | 0.086 | 2.1E-01 | 4.3E-01 |
| ab2363 | CEAM1 | CD66a | CEACAM1 | 10.42 | 0.010 | 9.5E-01 | 9.8E-01 |
| ab1577 | CEAM8 | CD66b | CEACAM8 | 9.78 | 0.037 | 4.3E-01 | 6.6E-01 |
| ab1629 | CEAM3,5 | CD66b | CEACAM3 | 7.80 | -0.085 | 4.0E-02 | 1.4E-01 |
| ab1635 | CEAM8 | CD66b | CEACAM8 | 7.60 | -0.040 | 3.9E-01 | 6.4E-01 |
| ab1633 | CEAM6 | CD66c | CEACAM6 | 8.30 | -0.007 | 8.9E-01 | 9.6E-01 |
| ab2714 | CEAM6 | CD66c | CEACAM6 | 7.55 | 0.007 | 8.9E-01 | 9.6E-01 |
| ab1630 | CEAM5 | CD66e | CEACAM5 | 8.15 | -0.065 | 2.1E-01 | 4.3E-01 |
| ab2720 | CEAM5 | CD66e | CEACAM5 | 8.54 | 0.069 | 1.4E-01 | 3.2E-01 |
| ab1470 | CD69 | CD69 | CD69 | 11.28 | 0.761 | 4.3E-06 | 3.8E-05 |
| ab1638 | CD69 | CD69 | CD69 | 8.11 | 0.103 | 1.6E-01 | 3.6E-01 |
| ab1639 | CD69 | CD69 | CD69 | 7.89 | 0.025 | 5.0E-01 | 7.3E-01 |
| ab1640 | CD69 | CD69 | CD69 | 8.32 | -0.007 | 8.4E-01 | 9.4E-01 |
| ab1370 | CD7 | CD7 | CD7 | 8.11 | -0.016 | 7.9E-01 | 9.1E-01 |
| ab1371 | CD7 | CD7 | CD7 | 9.15 | -0.646 | 8.4E-10 | 1.5E-08 |
| ab1571 | CD70 | CD70 | CD70 | 7.69 | -0.112 | 6.4E-02 | 1.9E-01 |
| ab1471 | TFR1 | CD71 | TFRC | 13.62 | -0.006 | 9.5E-01 | 9.8E-01 |
| ab1472 | TFR1 | CD71 | TFRC | 11.05 | -0.352 | 4.1E-06 | 3.8E-05 |
| ab1547 | TFR1 | CD71 | TFRC | 10.95 | -0.671 | 2.0E-09 | 3.2E-08 |
| ab2730 | TFR1 | CD71 | TFRC | 12.40 | -0.379 | 2.1E-07 | 2.1E-06 |
| ab1473 | CD72 | CD72 | CD72 | 9.18 | 0.080 | 1.7E-01 | 3.7E-01 |
| ab1474 | CD72 | CD72 | CD72 | 8.01 | 0.045 | 5.8E-01 | 7.9E-01 |
| ab1110 | 5NTD | CD73 | NT5E | 11.14 | 0.319 | 1.9E-03 | 1.0E-02 |
| ab1475 | CD79A | CD79a | CD79A | 8.54 | -0.016 | 8.1E-01 | 9.3E-01 |
| ab1476 | CD80 | CD80 | CD80 | 7.94 | -0.046 | 2.8E-01 | 5.3E-01 |
| ab2460 | CD80 | CD80 | CD80 | 7.56 | -0.014 | 8.5E-01 | 9.4E-01 |
| ab0993 | CD81 | CD81 | CD81 | 10.39 | 0.049 | 5.2E-01 | 7.4E-01 |
| ab1572 | CD81 | CD81 | CD81 | 10.25 | -0.173 | 5.0E-03 | 2.3E-02 |
| ab1477 | CD86 | CD86 | CD86 | 8.16 | 0.026 | 5.6E-01 | 7.7E-01 |
| ab1832 | UPAR | CD87 | PLAUR | 7.89 | 1.166 | 1.2E-11 | 2.7E-10 |
| ab1375 | CD8A | CD8a | CD8A | 9.02 | -0.023 | 7.7E-01 | 9.0E-01 |
| ab1376 | CD8A | CD8a | CD8A | 8.55 | 0.086 | 5.1E-01 | 7.4E-01 |
| ab1378 | CD9 | CD9 | CD9 | 10.96 | 0.389 | 3.7E-08 | 4.6E-07 |
| ab1531 | CD9 | CD9 | CD9 | 7.96 | 0.071 | 1.4E-01 | 3.2E-01 |
| ab1478 | TNR6 | CD95 | FAS | 11.13 | -0.111 | 7.4E-02 | 2.2E-01 |
| ab1899 | TNR6 | CD95 | FAS | 8.61 | -0.308 | 2.0E-05 | 1.7E-04 |
| ab1479 | AGRE5 | CD97 | CD97 | 9.60 | -0.006 | 9.9E-01 | 9.9E-01 |
| ab1830 | AGRE5 | CD97 | CD97 | 7.95 | 0.057 | 9.9E-02 | 2.6E-01 |
| ab1480 | 4F2 | CD98 | SLC3A2 | 9.76 | -0.325 | 5.6E-08 | 6.7E-07 |
| ab1481 | CD99 | CD99 | CD99 | 11.66 | -0.230 | 1.1E-07 | 1.2E-06 |
| ab3128 | CD99R | CD99 | CD99 | 8.30 | -0.081 | 6.2E-01 | 8.1E-01 |
| ab1401 | CDw17 | CDw17 | CDw17 | 8.07 | -0.051 | 2.3E-01 | 4.6E-01 |
| ab1493 | HLA-ABC | HLA-ABC |  | 10.35 | -0.708 | 3.2E-13 | 8.4E-12 |
| ab1496 | HLA-class II | HLA-class II |  | 8.17 | -0.066 | 3.0E-01 | 5.4E-01 |
| ab1495 | DPB1 | HLA-DP1B |  | 7.93 | -0.050 | 3.2E-01 | 5.6E-01 |
| ab1494 | HLA-DR | HLA-DR |  | 9.05 | 0.068 | 2.9E-01 | 5.4E-01 |

**4. Appendix References**

[1] M. Sill, C. Schr¨ oder, J. D. Hoheisel, A. Benner, and M. Zucknick. Assessment and optimisation of normalization methods for dual-colour antibody microarrays. BMC bioinformatics, 11:556. doi: 10.1186/1471-2105-11-556.

[2] Y. Benjamini and Y. Hochberg. Controlling the false discovery rate: A practical and powerful ap-

proach to multiple testing. Journal of the Royal Statistical Society: Series B (Methodological), 57(1):289– 300. doi: 10.1111/j.2517-6161.1995.tb02031.x.

[3] S. Yamada, N. Al-Sharabi, F. Torelli, AA. Volponi, L. Sandven, M. Ueda, I. Fristad, K. Mustafa. Harnessing the Antioxidative Potential of Dental Pulp Stem Cell-Conditioned Medium in Photopolymerized GelMA Hydrogels. Biomater Res. 2024 Sep 17;28:0084. doi: 10.34133/bmr.0084.
